# Achieving High Energy Efficiency: Recent Advances in Zn‐Air‐Based Hybrid Battery Systems

**DOI:** 10.1002/smsc.202300094

**Published:** 2023-11-27

**Authors:** Lei Yan, Jie Chen, Chen Yang, Jiqiang Ning, Yong Hu

**Affiliations:** ^1^ Xing Zhi College Zhejiang Normal University Jinhua 321004 China; ^2^ Key Laboratory of the Ministry of Education for Advanced Catalysis Materials Department of Chemistry Zhejiang Normal University Jinhua 321004 China; ^3^ Department of Optical Science and Engineering Fudan University Shanghai 200438 China; ^4^ College of Chemistry and Materials Engineering Zhejiang A&F University Hangzhou 311300 China

**Keywords:** oxygen evolution reactions, oxygen reduction reactions, redox reactions, working mechanisms, Zn-air hybrid batteries

## Abstract

Rechargeable Zn‐air batteries (ZABs) are regarded as an attractive green energy storage technology, featured with large theoretical energy densities and intrinsic high safety factors. However, hindered by the sluggish kinetics of both oxygen reduction reaction and oxygen evolution reaction, rechargeable ZABs are confronted with some critical challenges such as low operating voltage, poor energy efficiency, and limited cycle life. Zn‐air‐based hybrid batteries (ZAHBs), integrating the advantages of a conventional ZAB with supplementary redox reactions, have emerged as a promising solution to address those challenges. Based on working principles, the hybrid batteries can be categorized into two groups: Zn‐M/air hybrid batteries (M = Ni, Co, Ag, Cu, and Mn) and Zn‐X/air hybrid batteries (X = KI, ethanol, and urea), which can achieve improved energy efficiency and density by optimizing charge–discharge voltage. Herein, a comprehensive overview of ZAHBs is provided, including the classification, latest progress, and electrochemical properties, as well as detailed discussion of relevant mechanisms. Moreover, the perspectives and opportunities for future research in the field of hybrid battery systems are also outlined. This review shall give helpful guidance on the design and application of ZAHBs, and provide important insights into the development of new electrochemical energy storage systems.

## Graphical abstract

Zn‐air‐based hybrid batteries (ZAHBs) integrating the advantages of a conventional Zn‐air battery (ZAB) with supplementary redox reactions exhibit the reduced charge voltages, increased discharge voltages, as well as improved energy densities and efficiencies. The relevant mechanisms, classification, recent advances, and electrochemical performances of ZAHB systems are discussed in this review.

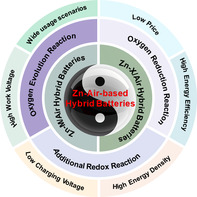

## 1 Introduction

Building a sustainable society depends on transitioning our energy usage away from fossil fuels and toward renewable energy sources.^[^ ^1^, ^2^, ^3^ ^]^ To successfully integrate power produced from diverse renewable energy sources into our everyday energy usage, electrochemical storage devices with high energy density and low cost are required.^[^ ^4^, ^5^, ^6^, ^7^, ^8^, ^9^ ^]^ Rechargeable metal‐air batteries utilize oxygen from the ambient air as one of the reactants, eliminating the need for dedicated oxygen storage, and enabling higher theoretical capacities solely determined by the metal electrodes.^[^ ^10^, ^11^, ^12^, ^13^, ^14^ ^]^ Taking lithium‐air battery (LAB) as an example, it possesses a super high theoretical capacity (3,862 Ah kg^−1^) and energy density (11 400 Wh kg^−1^, based on the voltage of 2.96 V).^[^ ^15^ ^]^ Nevertheless, LABs face safety concerns, given the usage of metallic lithium and aprotic electrolytes.^[^ ^16^ ^]^ In contrast, rechargeable Zn‐air batteries (ZABs) are a highly sought‐after research topic because of their superior safety and affordability brought about by the combination of Zn and aqueous electrolytes, as well as their high energy density (1,350 Wh kg^−1^) and theoretical capacity (820 mAh g_Zn_^−1^), making ZABs stronger in market competitiveness than LABs.^[^ ^17^, ^18^, ^19^ ^]^

**Scheme 1:**
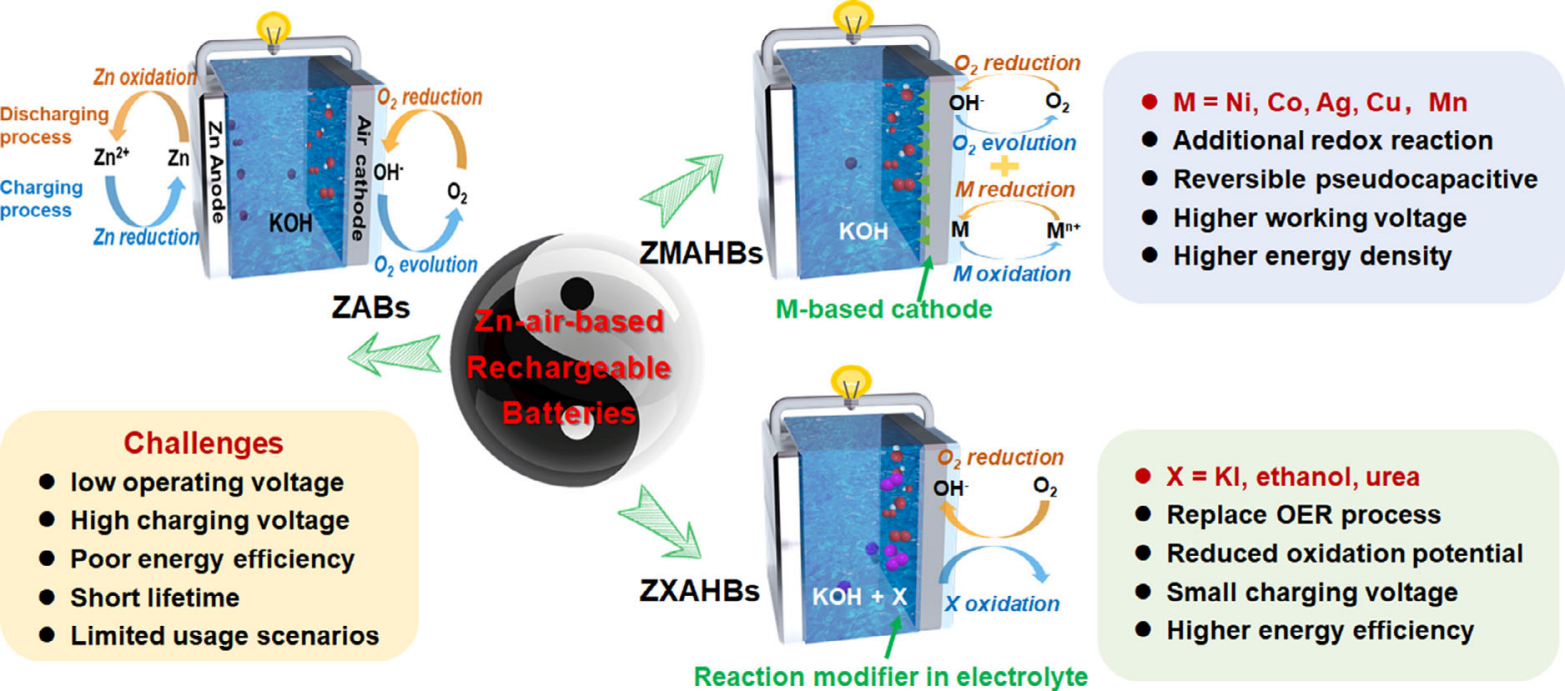
Schematic illustration of the battery structures and challenges/advantages of conventional ZABs, ZMAHBs, and ZXAHBs.

A conventional rechargeable ZAB consists of a metallic Zn anode, an aqueous electrolyte, and an air cathode electrode (**Scheme** **1**), and the battery undergoes oxygen reduction reaction (ORR) at cathode and Zn anode oxidation during discharging, while oxygen evolution reaction (OER) and Zn reduction happen in the meantime of charging process.^[^ ^20^, ^21^, ^22^, ^23^, ^24^ ^]^ Ranging from individual structural components to the entire system, ZABs encounter various problems in their practical applications. For instance, the lack of efficient and stable bifunctional electrocatalysts for the air electrode leads to high overpotentials for ORR and OER.^[^ ^25^, ^26^ ^]^ This undesirable behavior leads to a low actual operating voltage of ZABs (typically less than 1.20 V), and high charge voltage (higher than 2.0 V), consequently causing low practical capacity, and poor energy efficiency (usually below 65%).^[^ ^27^, ^28^, ^29^ ^]^ Besides, the high‐charging voltage induces oxidation of the cathode catalyst and speeds electrode degradation, aggravating the inactivation of active sites and shedding of active species, resulting in a decreased cycle life of battery.^[^ ^30^, ^31^, ^32^ ^]^ Additionally, ZABs operate as semiopen systems, which may accelerate the evaporation of the aqueous electrolyte, leading to a steep decline in capacity. The application scenarios for ZABs are limited due to the unsuitable for oxygen‐free or low‐oxygen environments such as sealed, vacuum, and underwater conditions.^[^ ^33^, ^34^, ^35^, ^36^ ^]^ Therefore, efficient methods to improve the energy efficiency and stability of ZABs and to expand their practical applications are urgently needed.

Closed rechargeable Zn‐M batteries (ZMBs, M = Ni, Co, Ag, Cu, etc.) use transition metal compounds such as metal oxide, hydroxides, or sulfide as the positive electrode materials, primarily utilizing aqueous alkaline electrolytes.^[^ ^37^, ^38^, ^39^ ^]^ The charging and discharging processes involve the reduction and oxidation of the Zn anode and the redox active material on the cathode electrode.^[^ ^40^ ^]^ This design provides several advantages, including a high working voltage and good theoretical energy density (≈1.8 V and 340 Wh kg^−1^ for Zn‐Ni battery).^[^ ^41^ ^]^ Unfortunately, the limitation of active material loading on positive electrodes in ZMBs hinders the increase of actual energy density.^[^ ^42^ ^]^ Additionally, the presence of the OER imposes a cut‐off voltage on ZMBs, limiting the full utilization of transition metal active sites and leading to reduced reversibility. Due to the structural similarity and performance complementarity, the integration of a half‐opened ZAB with a closed ZMB at the cell level emerges as a viable strategy to solve the above dilemmas.^[^ ^35^, ^43^ ^]^ Zn‐M/air hybrid batteries (ZMAHBs), combining ZABs with ZMBs at the cell level, which makes it possible to achieve both the high operating voltage characteristic of a ZMB (>1.7 V) and the large theoretical energy density associated with a ZAB (1,350 Wh kg^−1^) within a single ZMAHB.^[^ ^44^, ^45^ ^]^ Notably, the active materials used in the ZMBs can also function as catalysts for the oxygen electrocatalysis process in the ZABs, thus establishing a link between the electrochemical reactions of the two batteries and allowing high discharge voltage and energy density to be achieved simultaneously in ZMAHB.

Addressing the high thermodynamic equilibrium voltage (1.23 V) and the sluggish kinetics associated with the OER process during charging is another approach to improve the efficiency of ZABs. The slow OER behavior results in excessive charging voltage and electrocatalyst deactivation, which severely reduces the energy efficiency and cycle life of the battery. Therefore, the crucial key to enhancing the performance of ZABs lies in mitigating the OER overpotential and facilitating the charging process. Researchers have made significant efforts to improve OER kinetics and develop advanced catalysts that accelerate electron transfer, aiming to reduce the charging voltage.^[^ ^46^ ^]^ Nevertheless, the slow dynamics and high thermodynamic equilibrium voltage of OER impose an upper limit on ZABs, and the majority of reported studies have failed to exceed the charging voltage of ≈1.9 V. As an innovative solution to these challenges, Zn‐X/air hybrid batteries (ZXAHBs) (X = KI, ethanol and urea) have been proposed, in which easily oxidizable substances such as soluble potassium iodide (KI), ethanol, and urea are introduced into the electrolyte as reaction modifiers.^[^ ^47^, ^48^, ^49^ ^]^ By bypassing the slow OER pathway and employing a new oxidation reaction with accelerated kinetics and reduced oxidation potential, this novel hybrid battery system offers opportunities for lower charging voltages, improved energy efficiency, and enhanced cycle stability.

In this review, a comprehensive overview of ZAHBs is provided, focusing on their detailed discussions and configurations. Based on the working principle, two main groups of ZAHBs are identified: ZMAHBs (M = Ni, Co, Ag, Cu, and Mn) and ZXAHBs (X = KI, ethanol, and urea). The material properties, electrochemical performance, and potential applications of ZAHBs are summarized in detail. Furthermore, the challenges and opportunities of hybrid batteries in terms of electrode material optimization, electrolyte design, and comprehensive evaluation are also discussed. The intention of this work is to inspire future research on ZAHBs and provide a valuable reference for the design of novel electrochemical energy storage systems characterized by high energy density and efficiency.

## 2 Basic Cell Configurations and Working Mechanism of ZAHBs

**Figure 1:**
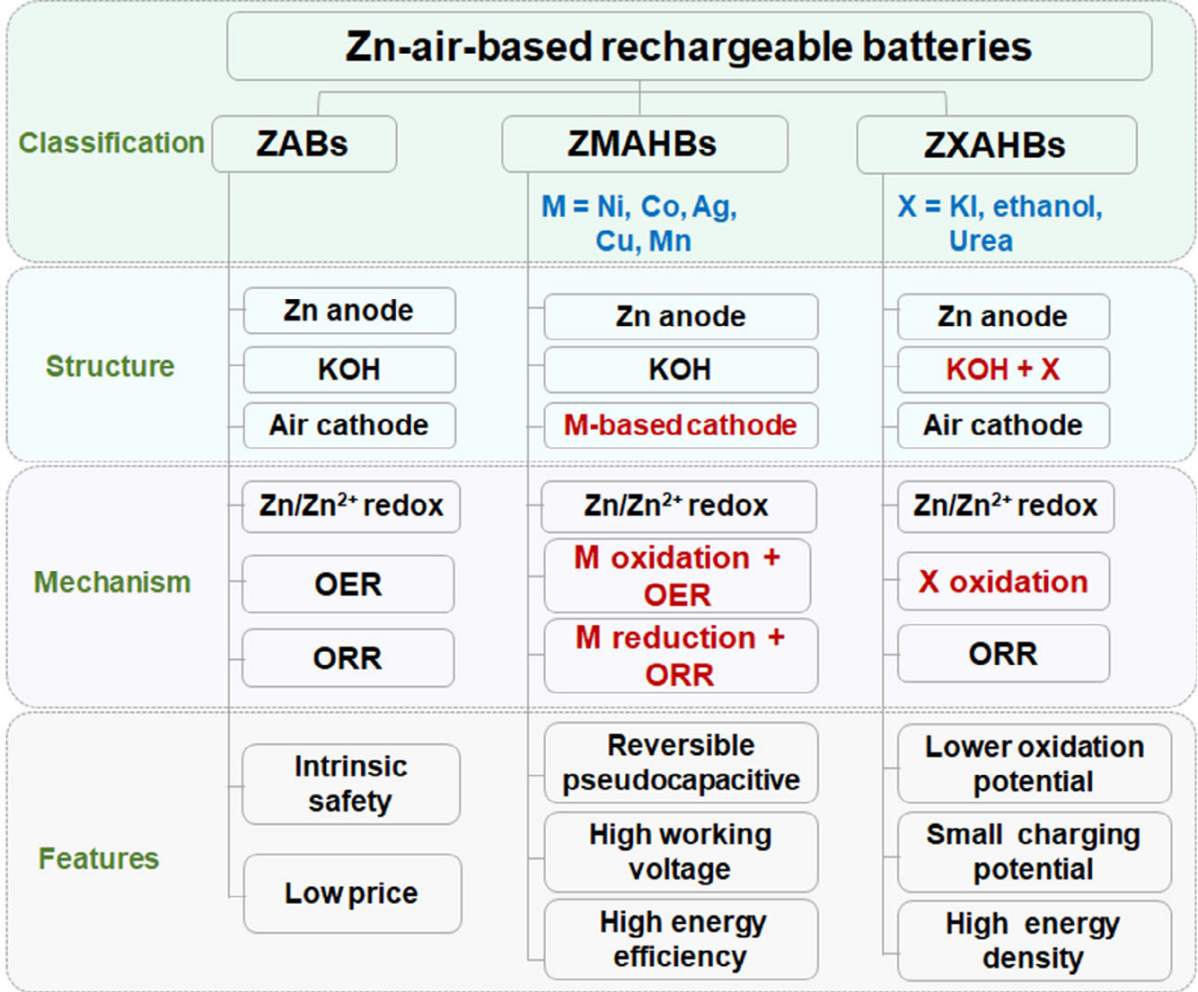
Classification, structures, working mechanisms, and features of the ZAHBs.

**Figure** **1** depicts the classification, working mechanism, and features of rechargeable ZAHBs. Both conventional ZAB and hybrid batteries consist of a Zn anode, an aqueous electrolyte, and an air cathode with active materials. In terms of operation, the Zn anode in ZAHB is fundamentally identical to that in conventional ZAB, where the oxidation of Zn and reduction of Zn(II) take place during discharge and charge cycles, respectively.^[^ ^50^ ^]^ This process can be described by Equation (1) under alkaline electrolytes.

Anode reaction:
(1)

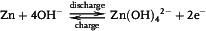

Generally, the electrochemical properties of ZMAHBs are manipulated by multifunctional cathode materials. M‐based catalysts are necessary and metal cation redox reactions are involved in the air cathode, which is different from the typical OER and ORR observed in a ZAB (as depicted in Equation (2)). Taking a Zn‐Ni/air hybrid battery as an example, in which Ni(OH)_2_ is employed as a multifunctional cathode catalyst.^[^ ^43^, ^51^ ^]^ The cathode undergoes electrocatalytic ORR and OER (Equation (2)) and faradaic redox reactions (Equation (3)). During discharging, the reduction of NiOOH occurs initially, followed by ORR. Conversely, during charging, the oxidation of Ni(OH)_2_ and OER takes place consecutively. The faradaic redox reaction (Equation (3)) generates reversible pseudocapacitive currents within a narrow potential range, leading to the hybrid battery exhibiting a higher discharge voltage and a lower charge voltage compared to the ZAB. Meanwhile, the electrocatalytic ORR (Equation (2)) provides a high discharge capacity and energy density by utilizing oxygen from the ambient air. It is important to note that although the redox reactions in ZMAHBs follow a similar principle of transition metal valence state changes, the specific reactions vary depending on the transition metal involved. For example, in the case of Co‐based catalysts, the product during the charging and discharging process is the redox reaction of Co_3_O_4_/CoOOH/CoO_2_. Similarly, Ag and Cu yield hypervalent oxides through redox reactions of Ag/Ag_2_O/AgO and Cu/Cu_2_O/CuO, respectively.^[^ ^52^, ^53^ ^]^ Detailed discussions of the electrode materials and their corresponding electrochemical properties are provided in the subsequent section.

Cathode reaction

(2) $$O_{2}+2H_{2}O+4e^{-}\overset{\text{disch}\arg e}{\underset{\text{ch}\arg e}{⇄}}4{\text{OH}}^{-}$$

(3) $${\text{NiOOH}+2H}_{2}{O+e}^{-}\overset{\text{discharge}}{\underset{\text{charge}}{⇄}}{\text{Ni}(\text{OH})}_{2}+{\text{OH}}^{-}$$

The ZXAHB has recently proposed using readily oxidized materials as reaction modifiers to substitute for the sluggish OER process. These reaction modifiers typically have a lower oxidation potential and superior oxidation thermodynamics. For instance, in Zn‐KI/air hybrid batteries, KI replaces OER process by facilitating the oxidation of I^−^ at a lower oxidation potential.^[^ ^54^ ^]^ The oxidation of I^−^ occurs through an electrochemical process, where I^−^ is initially oxidized to I_2_ via a 2e^−^ pathway, which has faster kinetics compared to the 4e^−^ pathway of OER (Equation (4)). Then, I_2_ disproportionates in the alkaline environment to form I^−^ and IO_3_^−^ spontaneously (Equation (5)).^[^ ^55^ ^]^ The oxidation product, IO_3_^−^, remains stable and is hardly reduced during the discharging process in the hybrid battery. Therefore, the ORR process continues during discharge, and the working principle at cathode can be described by Equation (6). By merit of the oxidation of I^−^ with accelerated kinetics and lower oxidation potential, a Zn‐KI/air hybrid battery can operate at significantly lower charging voltages with great energy efficiency.

KI‐modified cathodic oxidation reaction

(4) $${2I}^{-}-{2e}^{-}\overset{\text{charge}}{\to }I_{2}$$

(5)

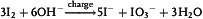




KI‐modified cathodic reduction reaction

(6) $$O_{2}+2H_{2}O+4e^{-}\overset{\text{discharge}}{\to }4{\text{OH}}^{-}$$

To elucidate the structure and reaction mechanism of ZAHBs, various techniques are employed to analyze and comprehend the morphology, composition, and electrochemical performance. The surface morphology of the electrodes can be studied using scanning electron microscopy (SEM) and transmission electron microscopy (TEM), which allow the identification of particle size, distribution, and surface structure from nano‐ to microscale.^[^ ^56^ ^]^ Additionally, X‐ray diffraction (XRD), X‐ray photoelectron spectroscopy (XPS), and Raman spectroscopy characterization tests are used to analyze the crystal structure and phase composition of the electrode materials. The dynamic changes of electrode and electrolyte during the charging and discharging process can be further monitored through continuous measurements of characterization techniques (e.g., XPS and Raman), which are crucial for understanding the mechanism of ZAHBs.^[^ ^51^, ^54^ ^]^ Furthermore, electrochemical tests including cyclic voltammetry (CV), linear sweep voltammetry (LSV), and galvanostatic charge–discharge (GCD), can be performed to evaluate the battery performance in terms of capacity, cycling stability, and charge/discharge voltage.^[^ ^57^ ^]^

## 3 The Type of ZAHBs

### 3.1 ZMAHBs

ZMAHBs, a hybrid system constructed by integrating ZMBs and ZABs in alkaline or neutral electrolytes, possess considerable characteristics such as high operating voltage, energy density, and long lifespan, making them attractive for potential large‐scale applications.^[^ ^58^ ^]^ Generally, the M‐based positive electrode materials employed in ZMBs determine their electrochemical parameters, including the discharge voltage platform and theoretical capacity, as shown in **Table** **1**.^[^ ^41^ ^]^ To exhibit the excellent performance of ZMAHBs, multifunctional electrocatalysts require abundant OER and ORR active sites and good pseudocapacitance. Considering the discharge voltage plateau of about 1.2 V and the charging plateau of about 2.0 V in ZABs, selecting a suitable oxide electron pair (**Table** **2**) in a ZMB system to improve the charge/discharge efficiency or extend the voltage range of the hybrid battery is necessary. Depending on the active metal component, ZMAHBs can be classified into several types (**Table** **3**), including Zn‐Ni/air, Zn‐Co/air, and Zn‐Ag/air hybrid batteries. This section will discuss recent advances in design concepts, material morphology, and electrochemical performance in the research of ZMAHBs.

**Table 1:** Performances of the ZMBs based on several typical positive electrode materials

Battery types	Positive electrode	Electrolyte	Energy density	Capacity	Discharge potential [V]	Power density	References
Zn‐Ni	Ni‐NiO/CC	6 m KOH + 0.5 m Zn(Ac)_2_	441.7 Wh kg^−1^	256 mAh g^−1^	1.6–1.8	41.6 kW kg^−1^	[59]
Zn‐Ni	Ni@Ni(OH)_2_ fiber	PVA gel with 1 m KOH	1.23 mWh cm^−3^	704 μAh cm^−3^	≈1.75	–	[60]
Zn‐Co	Co_3_O_4_@NF	6 m KOH + 0.1 m Zn(Ac)_2_	239 Wh kg^−1^	173.6 mAh g^−1^	≈1.7	–	[52]
Zn‐Co	Co_3_S_4_ nanosheets	3 m KOH + 0.1 m Zn(Ac)_2_	507 Wh kg^−1^	317 mAh g^−1^	1.6–1.65	19.2 kW kg^−1^	[82]
Zn‐Ag	MOF‐derived Ag nanowires	PVA gel with 1 m KOH‐saturated ZnO	1.87 mWh cm^−2^	1.245 mAh cm^−2^	≈1.5	2.8 mW cm^−2^	[110]
Zn‐Ag	CNTF‐NCA Ag_2_O@PEDOT:PSS	PVA gel with 1 m KOH‐saturated ZnO	1.57 mWh cm^−2^	1.05 mAh cm^−2^	≈1.5	14.4 mW cm^−2^	[113]
Zn‐Cu	Cu clusters	1 m KOH	–	718 mAh g^−1^	≈0.76	–	[122]
Zn‐Cu	Cu@C	45 wt% KOH	60 Wh L^−1^	154 mAh g^−1^	≈0.8	–	[121]
Zn‐Mn	α‐MnO_2_@graphene	2 m ZnSO_4_ + 0.2 m MnSO_4_	406.6 Wh kg^−1^	382.2 mAh g^−1^	≈1.33	–	[125]
Zn‐Mn	MnO_*x*_ nanoparticles	1 m ZnSO_4_ + 0.3 m MnSO_4_	845.1 Wh kg^−1^	133.3 mAh g^−1^	1.53 and 1.55	1212 W kg^−1^	[126]

**Table 2:** The voltage range of different oxidation electron pairs at ZMAHBs

Battery types	Oxidation electron pairs	Voltage range (vs Zn) [V]
Zn‐Ni/air	Ni^2+^/Ni^3+^	1.7–1.9
Zn‐Co/air	Co^2+^/Co^3+^	1.5–1.75
	Co^3+^/Co^4+^	1.75–2
Zn‐Ag/air	Ag/Ag^+^	1.6–2.0
	Ag^+^/Ag^2+^	2.0–2.1
Zn‐Cu/air	Cu/Cu^+^	0.9–1.2
	Cu^+^/Cu^2+^	1.7–1.9
Zn‐Mn/air	Mn^2+^/Mn^4+^	1.2–1.6

**Table 3:** Performances of the ZMAHBs based on different cathode materials

Battery types	Cathode materials	Electrolyte	Discharge voltage [V]	Charge voltage [V]	Capacity@j	Energy density@j	Power density@j	Cycling stability@j	Energy efficiency	References
Zn‐Ni/air	O‐NiO@MCSN	–	1.12	1.72	800.3 mAh g^−1^@1 mA cm^−2^	961 Wh kg^−1^@1 mA cm^−2^	33.9 mW cm^−2^@20 mAcm^−2^	–	–	[79]
Zn‐Ni/air	Pt‐Ni(OH)_2_	6 m KOH + saturated ZnO	1.74/1.25	1.94	–	–	98.1 mW cm^−2^@140 mA cm^−2^.	1800 min@2 mA cm^−2^	85%	[64]
Zn‐Ni/air	Ni_3_S_2_@SNCNT	6 m KOH + 0.2 m Zn(OAc)_2_	1.2	1.64/2.1	809 mAh g^−1^@2 mA cm^−2^	963 Wh kg^−1^@2 mA cm^−2^	85.1 mW cm^−2^@89 mA cm^−2^	150 h@5 mA cm^−2^	73%	[74]
Zn‐Ni/air	Ni_0.88_Fe_0.12_LDH	6 m KOH + 0.2 m Zn(OAc)_2_	1.7/1.2	1.85/2.0	–	–	120 mW cm^−2^@200 mA cm^−2^	67 h@4 mA cm^−2^	87%	[63]
Zn‐Ni/air	Ni_3_S_2_	6 m KOH + 0.2 m Zn(OAc)_2_	1.65/1.1	1.9/2.1	–	1105 Wh kg^−1^	60 mW cm^−2^@40 mA cm^−2^	300 h	–	[69]
Zn‐Co/air	Co_3_O_4−*x*_	6 m KOH + 0.2 m Zn(OAc)_2_	1.23	1.93/2	800 mAh g^−1^@2 mA cm^−2^	1060 Wh kg^−1^@2 mA cm^−2^	3200 W kg^−1^@80 mA cm^−2^	420 h@5 mA cm^−2^	63%	[101]
Zn‐Co/air	Co_3_O_4_	6 m KOH + 0.2 m Zn(OAc)_2_	1.3/1.05	1.8/2.0	805 mAh g^−1^@1 mA cm^−2^	936 Wh kg^−1^@1 mA cm^−2^	38.6 mW cm^−2^@25 mA cm^−2^	26 h@10 mA cm^−2^	45%	[97]
Zn‐Co/air	Co_3_O_4−*x*_	6 m KOH + 0.2 m Zn(OAc)_2_	1.84/1.2	1.92/2.02	805.7 mAh g^−1^@3 mA cm^−2^	961 Wh kg^−1^@3 mA cm^−2^	84.2 mW cm^−2^@80 mA cm^−2^	800th cycle@3 mA cm^−2^	70.3%	[90]
Zn‐Co/air	Co_3_O_4_/Ni foam	6 m KOH + 0.1 m Zn(OAc)_2_	1.6/1.06	2.25	771 mAh g^−1^@5 mA cm^−2^	–	35.7 mW cm^−2^@68 mA cm^−2^	333 h@10 mA cm^−2^	70%	[52]
Zn‐Co/air	NiO@Co_3_S_4_	6 m KOH + 0.2 m Zn(OAc)_2_	1.75/1.28	1.8/1.95	–	–	26.2 mW cm^−2^@49 mA cm^−2^	200 h@2 mA cm^−2^	–	[103]
Zn‐Co/air	Co_3_O_4_	6 m KOH + 0.2 m Zn(OAc)_2_	1.85/1.0	1.91/2	792 mAh g^−1^@1 mA cm^−2^	–	41 mW cm^−2^@24 mA cm^−2^	100 h@1 mA cm^−2^	52.3%	[87]
Zn‐Ni‐Co/air	NiCo_2_O_4−*δ*_@C	6 m KOH + 0.2 m Zn(OAc)_2_	1.2	1.85	785 mAh g^−1^	950 Wh kg^−1^	81.4 mW cm^−2^@80 mA cm^−2^	5000th cycle@6 mA cm^−2^	64.9%	[106]
Zn‐Ni‐Co/air	NF@Co_3−*x*_Ni_*x*_O_4_/Co_3_O_4_	6 m KOH + 0.2 m Zn(OAc)_2_	1.67/1.1	1.38/1.82	812 mAh g^−1^@5 mA cm^−2^	922 Wh kg^−1^@5 mA cm^−2^	–	600 h@20 mA cm^−2^	62.2%	[104]
Zn‐Ni‐Co/air	MnS‐Ni_*x*_Co_1−*x*_S_2_	6 m KOH + 0.2 m Zn(OAc)_2_	1.7/1.3	1.96	–	–	153 mW cm^−2^@100 mA cm^−2^	330 h@5 mA cm^−2^	69%	[96]
Zn‐Ni‐Co/air	NiCoSe_2_@NiOOH/CoOOH	6 m KOH + 0.2 m Zn(OAc)_2_	1.7/1.0	1.8/2.0	–	944.8 Wh kg^−1^	–	100 h@4 mA cm^−2^	50%	[105]
Zn‐Ag/air	Ag/stainless steel wire screen	6 m KOH + 0.2 m Zn(OAc)_2_	1.8/1.54/1.1	1.80/1.98	–	–	–	551 h@20 mA cm^−2^	85%	[40]
Zn‐Ag/air	AgNBs	6 m KOH + 0.2 m Zn(OAc)_2_	1.2	1.66/2	–	–	82.2 mW cm^−2^@65 mA cm^−2^	–	76.9%/60%	[116]
Zn‐Ag/air	HEO/Ag_0.3_	6 m KOH + 0.2 m Zn(OAc)_2_	1.7/1.2	1.75/2.0	810 mAh g^−1^@10 mA cm^−2^	–	131 mW cm^−2^@230 mA cm^−2^	450 h@2 mA cm^−2^	65%	[120]
Zn‐Ag/air	Ag + RuO_2_/CNT	6 m KOH + 0.2 m Zn(OAc)_2_	1.2	1.6/1.85	800 mAh g^−1^@2 mA cm^−2^	944 Wh kg^−1^@2 mA cm^−2^	91.9 mW cm^−2^@157 mA cm^−2^	20 h@10 mA cm^−2^	75%/65%	[115]
Zn‐Cu/air	CuO	6 m KOH + 0.2 m Zn(OAc)_2_	1.28/1.13/0.79	1.93	693.7 mAh g^−1^	–	–	1500 cycles	–	[123]
Zn‐Ni‐Cu/air	Cu_*X*_O@NiFe‐LDH||Co‐N‐C dodecahedrons	6 m KOH + 0.2 m Zn(OAc)_2_	1.8/1.27	1.88/1.95	824 mAh g^−1^	940 Wh kg^−1^	177 mW cm^−2^@320 mA cm^−2^	500 h@10 mA cm^−2^	79.6%	[124]
Zn‐Mn/air	PMO/N‐rGO	2 m NH_4_Cl + 0.2 m ZnCl_2_ + 0.02 m MnSO_4_	1.4/0.9	1.72/2.1	488.6 mAh g^−1^@1 mA cm^−2^	420 Wh kg^−1^@1 mA cm^−2^	7.3 mW cm^−2^@7 mA cm^−2^	1350 h@1 mA cm^−2^	63.2%	[127]

#### 3.1.1 Zn‐Ni/Air Hybrid Batteries

**Figure 2:**
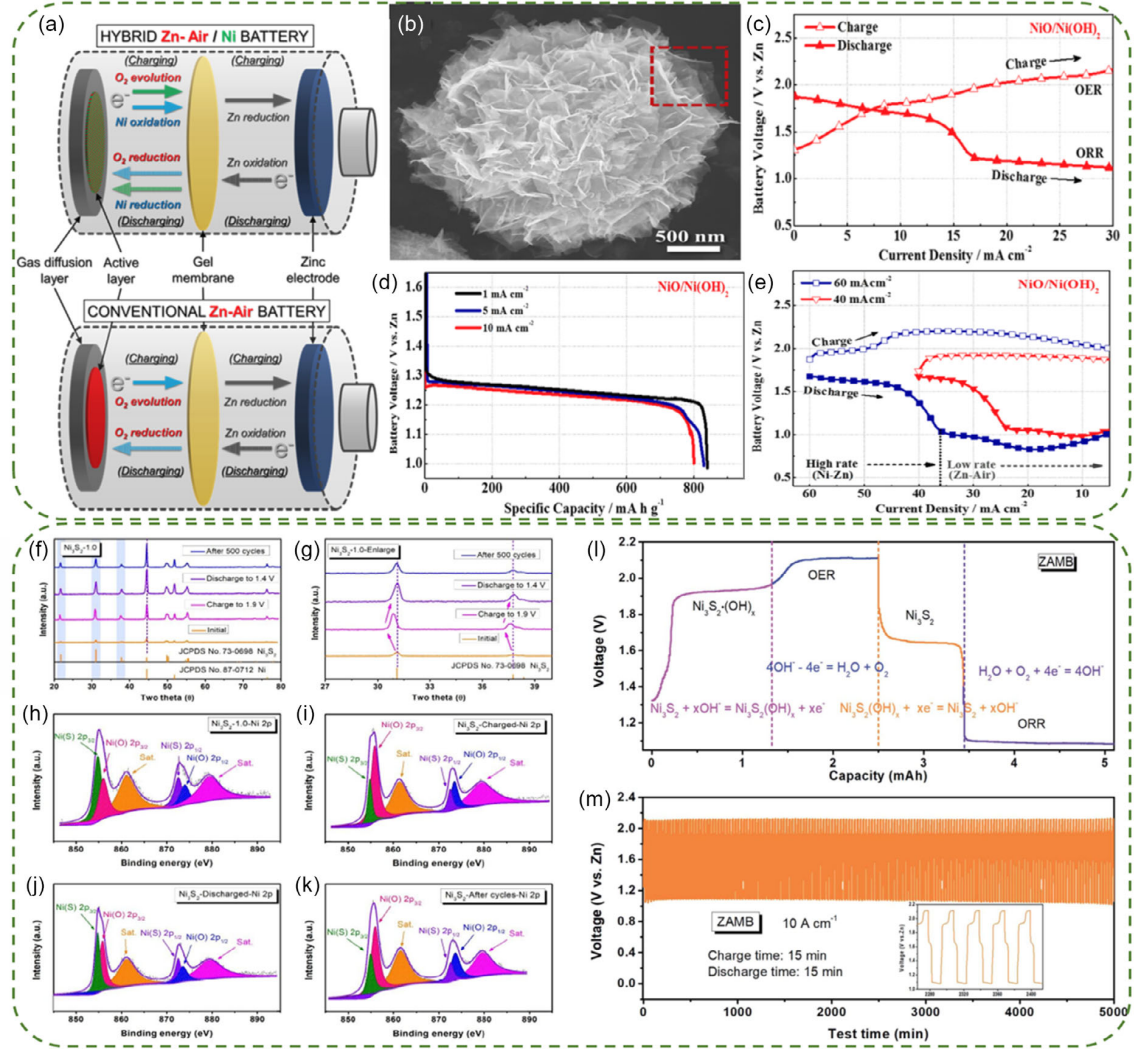
a) Schematic representation of a Zn‐Ni/air hybrid battery (top) and a conventional ZAB (bottom). b) SEM image of NiO/Ni(OH)_2_ mesoporous spheres. c) GCD voltage behavior, d) full discharge profiles obtained at current densities of 1, 5, and 10 mA cm^−2^, and e) reversed current rate galvanodynamic charge and discharge profiles of NiO/Ni(OH)_2_‐based battery. Reproduced with permission.^[^ ^43^ ^]^ Copyright 2016, American Chemical Society. f) XRD patterns and g) partially enlarged details of Ni_3_S_2_ in different states. High‐resolution Ni 2p spectrum of Ni_3_S_2_‐1.0: h) initial, i) charged to 1.9 V, j) discharged to 1.4 V, and k) after 100 cycles. l) GCD curve and m) cycling tested at 10 mA cm^−2^ of Zn‐Ni/air hybrid battery based on Ni_3_S_2_.^[^ ^69^ ^]^ Copyright 2019, Royal Society of Chemistry.

Ni‐based materials, such as NiO and Ni(OH)_2_, are highly favored for hybrid batteries due to their multiple valance states and high electrochemical activity.^[^ ^59^, ^60^ ^]^ A typical alkaline Zn‐Ni battery using Ni(OH)_2_ as electrode material shows an open‐circuit voltage (OCV) of ≈1.75 V and a theoretical energy density of 340 Wh kg^−1^.^[^ ^61^, ^62^ ^]^ Consequently, the integration of Ni‐based catalysts into ZMAHBs represents the primary research direction currently. For example, Chen et al. were the first to demonstrate the integration of two features in a Zn‐Ni/air hybrid battery using NiO/Ni(OH)_2_ nanoflakes as the active material (**Figure** **2**a).^[^ ^43^ ^]^ NiO/Ni(OH)_2_ processes abundant OER and ORR active sites and favorable electrochemical properties such as pseudocapacitance. These properties allow for faster charge transfer and enhanced material utilization. The SEM image (Figure 2b) shows that NiO/Ni(OH)_2_ sample self‐assembled into highly rippled spherical structures, effectively preventing nanoflakes from stacking and increasing the specific surface area. During the charging process (Figure 2c), two voltage platforms were observed in the NiO/Ni(OH)_2_‐based hybrid battery. The first plateau near 1.7 V could be attributed to the oxidation of Ni^2+^ to Ni^3+^ (NiO/Ni(OH)_2_ → NiOOH), while the second plateau ≈2.1 V corresponded to OER process. The discharge plateau near 1.7 V was associated with the reduction process of active Ni species (NiOOH → NiO/Ni(OH)_2_) and 1.1 V corresponds to the ORR process. Additionally, the NiO/Ni(OH)_2_‐based hybrid battery exhibited a higher discharge voltage of 1.3–1.2 V and a more competitive specific discharge capacity of 800 mAh g^−1^ at 10 mA cm^−2^ (Figure 2d), compared to traditional ZAB (<1.2 V, <600 mAh g^−1^). At low current density, the charge and discharge curves only showed one plateau, which may be caused by the overlapping of the relatively smaller OER overpotential with the fast kinetics of Ni oxidation (Figure 2e). As the current density increased, a new plateau appeared, which could be attributed to the repaid oxidation of the active Ni species. Consequently, the NiO/Ni(OH)_2_‐based hybrid battery exhibited high rate performance and power density (gravimetric, 2,700 W kg^−1^; volumetric, 14 000 W L^−1^). Askari et al. utilized a hydrothermal method to introduce varying amounts of Fe into α‐Ni(OH)_2_ to form a stable layered double hydroxide (NiFe‐LDH),^[^ ^63^ ^]^ which shows significantly enhanced redox conversion capacity and good reversibility. As a result, the hybrid battery based on NiFe‐LDH cathode materials exhibited an energy conversion efficiency of 87% at 4 mA cm^−2^, and a peak power density of 100 mW cm^−2^. Moreover, Pt with Ni(OH)_2_ electrocatalysts was also used as a cathode to construct a Zn‐Ni/air hybrid battery in a pouch‐type cell with a lean electrolyte.^[^ ^64^ ^]^ This hybrid battery exhibited an energy efficiency of 85% and demonstrated a prolonged cycle life of 100 cycles at 2 mA cm^−2^, surpassing the performance of Zn‐Ni battery (54% energy efficiency, 50 cycles).

Compared to Ni‐based oxides/hydroxides, transition metal sulfides with large interlayer spacing and high reversible redox properties have been proposed as potential catalysts for efficient HAZBs.^[^ ^65^, ^66^, ^67^, ^68^ ^]^ Huang et al. proposed the use of interconnected and distorted ultra‐thin Ni_2_S_3_ nanosheets as a cathode material for a Zn‐Ni/air hybrid battery.^[^ ^69^ ^]^ Ex‐XRD patterns were carried out to investigate the energy storage mechanism, revealing that the diffraction peak of Ni in the Ni_3_S_2_‐1.0 sample remained unchanged (Figure 2f). In the charged state, the diffraction peaks of Ni_3_S_2_ moved to a low‐angle region, indicating a significant deviation, while the peaks after 500 cycles shifted slightly toward a low‐angle direction (Figure 2g). The lack of noticeable shifts in other states may be attributed to the insertion of OH^−^ and the subsequent formation of Ni_3_S_2_(OH)_*x*_. The presence of intensified Ni‐O peaks in the XPS spectrum during charging further confirmed the transformation of Ni_3_S_2_·(OH)_*x*_ (Ni_3_S_2_ + *x*OH^−^ ↔ Ni_3_S_2_·(OH)_*x*_ + *x*e^−^) (Figure 2h–k). During discharging process, the decrease in Ni‐O peaks demonstrated the reversibility of the transformation. Consequently, the Zn‐Ni/air hybrid battery using Ni_3_S_2_ nanosheets exhibited two stages in GCD curves at 1.7 and 1.1 V, representing the formation of Ni_3_S_2_·(OH)_*x*_ and reformation of Ni_3_S_2_, respectively (Figure 2l). This hybrid battery delivered an energy density of 1,105 Wh kg^−1^ and long‐term stability of over 5,000 min at 10 mA cm^−2^ (Figure 2m).

**Figure 3:**
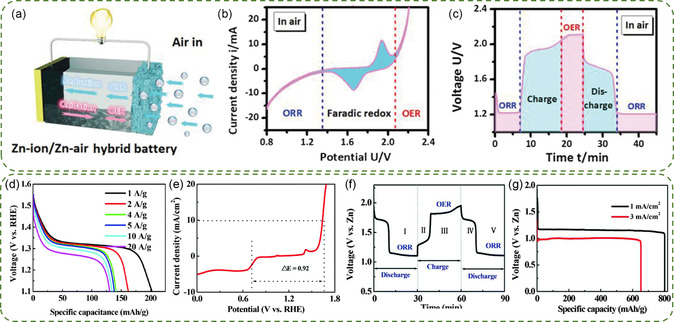
a) Schematic illustrations of Zn‐Ni/air hybrid battery, b) CV curves, and c) GCD curves of hybrid battery based on Ni_3_S_2_@SNCNT working in the air‐exposure conditions. Reproduced with permission.^[^ ^74^ ^]^ Copyright 2022, Elsevier. d) The discharge curve under various current densities, e) ORR and OER activities of O‐NiO@MCSN. f) Discharge–charge–discharge profiles and g) specific capacity of Zn‐Ni/air hybrid battery based on O‐NiO@MCSN. Reproduced with permission.^[^ ^79^ ^]^ Copyright 2020, Royal Society of Chemistry.

The limitations of pristine transition metal oxide/hydroxides and transition metal sulfides, such as poor electrical conductivity, low surface area and instability, continue to limit their practical utilization.^[^ ^70^ ^]^ To overcome these challenges, modification strategies have been proposed to integrate transition metal compounds with carbonaceous substrates, aiming to achieve improved physicochemical characteristics.^[^ ^71^, ^72^, ^73^ ^]^ Deng et al. successfully constructed a hollow fiber composed of heterostructures of S, N‐codoped carbon nanotubes confined Ni sulfides (Ni_3_S_2_@SNCNT).^[^ ^74^ ^]^ In the presence of O_2_‐saturated environment, the hybrid battery with Ni_3_S_2_@SNCNT hollow fiber cathode (**Figure** **3**a) demonstrated strong peaks associated with the OER and ORR at high voltage (≥2.05 V) and low voltage (≤1.35 V), respectively (Figure 3b). The one pair of faradaic redox peaks in the middle potential range (1.35–2.05 V) corresponded to the faradaic redox process of Ni^2+^/Ni^3+^ via the reaction of Ni_3_S_2_ + *x*OH^−^ ↔ Ni_3_S_2_(OH)_*x*_ + *x*e^−^. Moreover, the GCD profiles (Figure 3c) revealed the coexistence of two sets of electrochemical reactions, namely, the electrocatalytic ORR/OER and the faradaic redox reaction. As a result, the hybrid battery showed an energy density of 963 Wh kg^−1^ and good durability with an energy efficiency of ≈73% of 230 cycles at 5 mA cm^−2^.

The enhancement of electrocatalytic activity can be achieved through the creation of defects, which play a crucial role in catalysis due to their abundant local electrons, which promote the effective adsorption and activation of O_2_.^[^ ^75^, ^76^ ^]^ Among these defects, oxygen vacancies (O_V_) are the most commonly observed anion vacancy (point) defects.^[^ ^77^, ^78^ ^]^ Oxygen vacancy‐rich NiO with S, N comodified mesoporous carbon was used as the cathode material, exhibiting good pseudocapacitance properties and promising ORR/OER performance (Figure 3d,e).^[^ ^79^ ^]^ These advantages make it an ideal cathode material for a Zn‐Ni/air hybrid battery, as evidenced by the presence of two discharge voltage plateaus at 1.72 and 1.12 V (Figure 3f). Additionally, under a discharge current density of 1 mA cm^−2^, the specific capacity and energy density of hybrid battery reached 800.3 mAh g^−1^ and 961 Wh kg^−1^, respectively (Figure 3g).

#### 3.1.2 Zn‐Co/Air Hybrid Batteries

**Figure 4:**
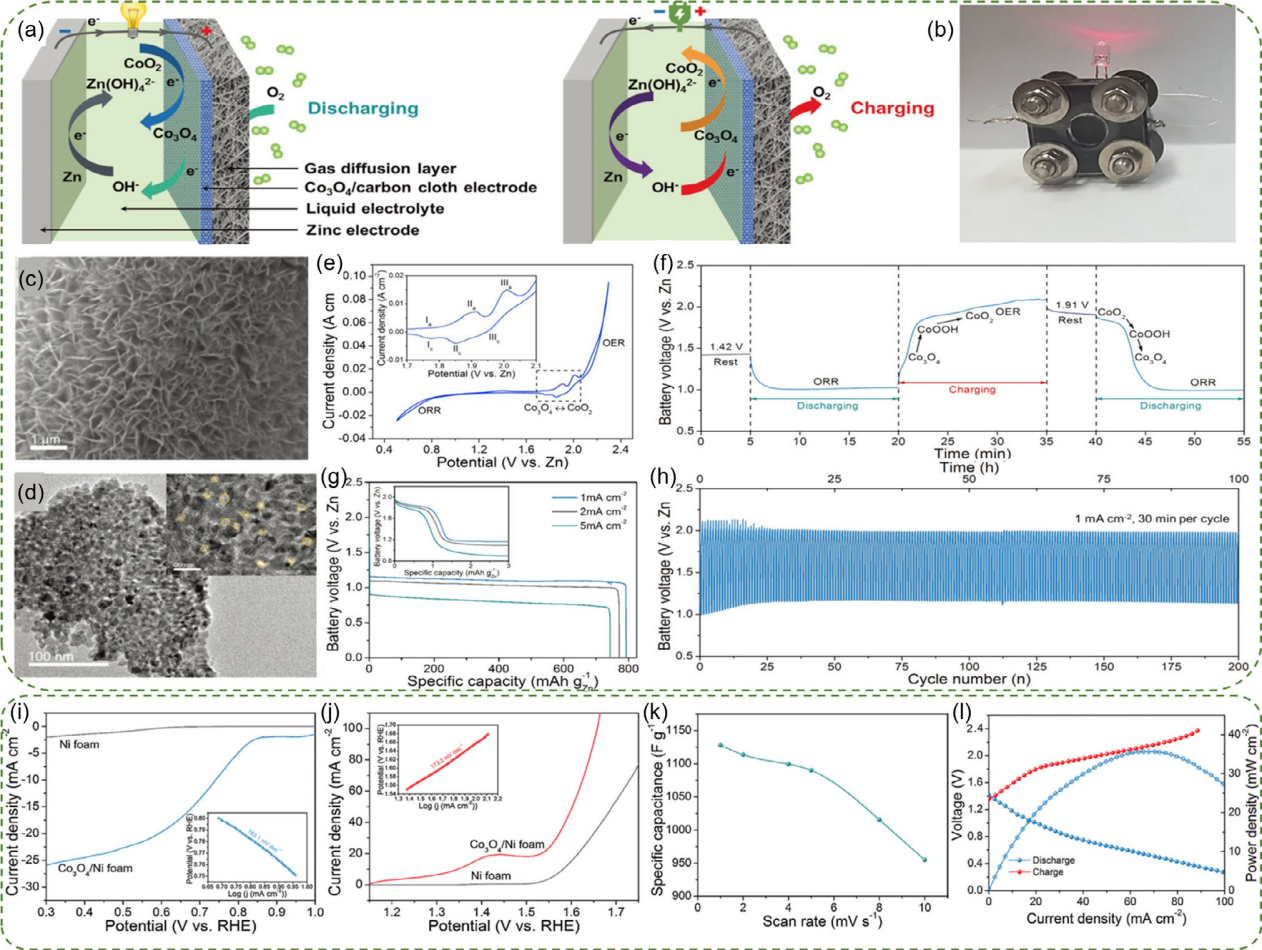
a) Schematic diagram of the Zn‐Co/air hybrid battery with the designed electrochemical processes during discharging and charging. b) Photograph of the homemade hybrid battery, a red LED (1.8 V) can be lit up. c) SEM images and d) TEM images of the Co_3_O_4_ nanosheets. e) The CV test, f) discharging and charging curves, and g) full discharge voltage profiles at current densities of 1, 2, and 5 mA cm^−2^. Inset: the voltage profiles of Zn‐Co_3_O_4_ reaction. h) Long‐term cycling stability at 1 mA cm^−2^. Reproduced with permission.^[^ ^87^ ^]^ Copyright 2018, John Wiley and Sons. i) ORR and j) OER LSV curves and the Tafel plots of Co_3_O_4_/Ni foam. k) The specific capacitance according to CV curves of Co_3_O_4_/Ni foam electrode in 0.1M KOH aqueous electrolyte. l) Polarization curves and the corresponding power density of Zn‐Co/air hybrid battery with the Co_3_O_4_/Ni foam electrode. Reproduced with permission.^[^ ^52^ ^]^ Copyright 2019, Elsevier.

Cobalt‐based materials (e.g., CoO and Co_3_O_4_) have attracted attention for their good electrochemical redox activity.^[^ ^80^, ^81^, ^82^ ^]^ The Co_3_O_4_‐based alkaline Zn‐Co battery processes a theoretical capacity of 446 mAh g^−1^ due to its ability to oxidize to Co^4+^.^[^ ^83^, ^84^ ^]^ Besides, cobalt oxides have been used as electrocatalysts in ZABs because of their ease of preparation and high catalytic activities.^[^ ^85^, ^86^ ^]^ Tan et al. synthesized vertical interconnected arrays of Co_3_O_4_ nanoflakes grown directly on carbon cloth, as depicted in **Figure** **4**a,b.^[^ ^87^ ^]^ The SEM and TEM images revealed that each nanosheet was composed of crystalline Co_3_O_4_ nanoparticles with abundant mesoporous (Figure 4c,d). The CV, performed in 6 m KOH, revealed three sets of redox peaks in the region of 1.7 and 2.1 V (Figure 4e). The first pair (I_a_/I_c_) corresponded to the electrochemical transition involving Co(OH)_2_ with Co_3_O_4_ species (3Co(OH)_2_ + 2OH^−^ ↔ Co_3_O_4_ + 4H_2_O + 2e^−^). The second (II_a_/II_c_) and the third (III_a_/III_c_) redox pairs could be ascribed to the conversion between Co_3_O_4_ to CoOOH and CoOOH to CoO_2_ species, respectively (Co_3_O_4_ + OH^−^ + H_2_O ↔ 3CoOOH + e^−^; CoOOH + OH^−^ ↔ CoO_2_ + H_2_O + e^−^). This observation was different from the Zn‐Ni/air hybrid battery with only one redox couple (Ni^2+^/Ni^3+^).^[^ ^88^ ^]^ The occurrence of multiple redox reactions within a narrow potential window was beneficial for improving the discharge voltage of the battery. The assembled Zn‐Co/air hybrid battery was further tested under ambient air. In the first discharging (Figure 4f), the steady and flat voltage plateau at 1.0 V represents the ORR. Subsequently, during the charging, two processes take place, demonstrating the oxidation of Co_3_O_4_ (Co_3_O_4_ → CoOOH → CoO_2_) and the following OER. In the second discharging, two voltage platforms were displayed: the first platform ≈1.85 V represented the reduction of Co (CoO_2_ → CoOOH → Co_3_O_4_), and the second plateau ≈1.0 V stood for ORR process. The discharging capacity was 792 mAh g_Zn_^−1^ at 1 mA cm^−2^ (Figure 4g).^[^ ^89^ ^]^ Furthermore, the hybrid battery could be continuously cycled for more than 100 h (Figure 4h) and maintained stable voltages of 1.15 and 1.98 V over 200 cycles. To further enhance performance, the porous Co_3_O_4_ nanowires were directly grown on Ni foam, resulting in a significant reduction in interfacial resistance and an improvement in electrical conductivity.^[^ ^52^ ^]^ The resulting Co_3_O_4_/NF achieved comparable electrochemical performance (Figure 4i,j), including a high limiting current density of −25.8 mA cm^−2^ for ORR at 0.3 V, and a high current density of 40 mA cm^−2^ at 1.58 V for OER. Furthermore, owing to efficient diffusion of electrolyte ions across the available electrode surfaces, the catalyst exhibited a high capacitance value of 1,128 F g^−1^ at a low scan rate of 1 mV s^−1^ (Figure 4k). In terms of assembled Zn‐Co/air hybrid battery delivered a peak power density of 35.7 mW cm^−2^ and a prolonged operational lifetime of 1,000 cycles at 10 mA cm^−2^ (Figure 4l).

**Figure 5:**
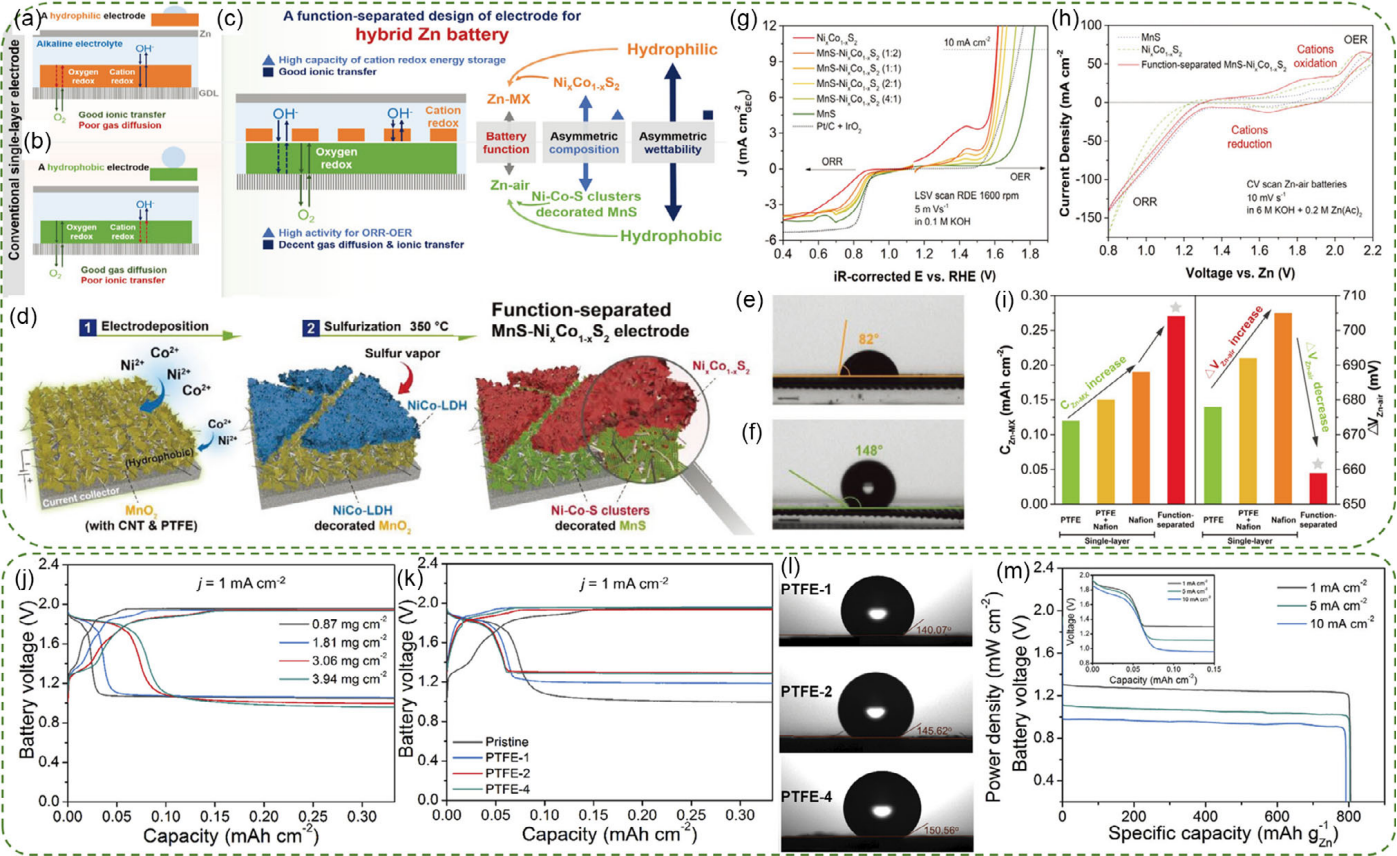
Conventional single‐layer electrodes with a) a hydrophilic surface and b) a hydrophobic surface. c) The advantages in different layers of a function‐separated electrode. d) Illustration of the preparing process. Wettability characteristics on e) hydrophilic NixCo_1−*x*_S_2_ and f) hydrophobic MnS. g) ORR and OER polarization curves of MnS, NixCo_1−*x*_S_2_, and their physical mixture compared with Pt/C + IrO_2_. h) CV scan profiles of the hybrid battery with MnS‐Ni_*x*_Co_1−*x*_S_2_ compared to electrodes with only MnS or NixCo_1−*x*_S_2_. i) Comparison of battery performance based on the two essential parameters. Reproduced with permission.^[^ ^96^ ^]^ Copyright 2020, John Wiley and Sons. j) Charge–discharge voltages of hybrid battery with Co_3_O_4_/carbon cloth of different Co_3_O_4_ loadings at 1 mA cm^−2^. k) Charge–discharge voltage profiles of the electrodes with different PTFE loadings at 1 mA cm^−2^. l) Contact angles of water droplets on PTFE‐1, PTFE‐2, and PTFE‐4 electrode surfaces. m) Full discharge voltage profiles at 1, 5, and 10 mA cm^−2^. Reproduced with permission.^[^ ^97^ ^]^ Copyright 2018, Elsevier.

The unsatisfactory utilization ratio of Co_3_O_4_ in alkaline Zn‐Co battery results in a low discharge capacity, while its mediocre performance toward oxygen electrocatalysis leads to low power density and energy density in ZAB.^[^ ^90^ ^]^ To address these challenges and enhance the performance of hybrid batteries, researchers have explored various strategies, including morphology and microstructure optimization, surface state modification, and introduction of additional elements.^[^ ^84^, ^91^, ^92^, ^93^, ^94^, ^95^ ^]^ A function‐separated design strategy was developed by the Shao group, which allocated the two battery functions to the two sides of the cathode (**Figure** **5**a–c).^[^ ^96^ ^]^ The cathode electrode consisted of a hydrophobic MnS decorated with Ni*‐*Co‐S nanoclusters, enabling smooth gas diffusion and electrocatalytic activity for both ORR and OER. Additionally, a hydrophilic Ni_*x*_Co_1−*x*_S_2_ facilitated the rapid ionic transfer and exhibited superior performance for Zn‐Co battery (Figure 5d–f). As a result, the hybrid battery with the function‐separated electrode displayed a high short‐term discharge voltage of ≈1.7 V, a power density of 153 mW cm^−2^, a good round‐trip efficiency of 75%, and cycling stability of 330 h (Figure 5g–i).

Ni et al. investigated the loading of active material and surface hydrophobicity of cathode electrodes to enhance the activity of hybrid batteries.^[^ ^97^ ^]^ The results indicated that the capacity of the Zn‐Co/air hybrid battery significantly increases with loading, but this improvement becomes limited with further increases (Figure 5j). This limitation arises from the fact that while both active material and surface area increase, the enlarged dimension of the nanosheets introduces transport resistance. In the case of a specific electrode, an increase in hydrophobicity initially raises the discharge voltage of the ZAB by creating a pathway for gaseous oxygen transport. However, the voltage subsequently reduces owing to the coverage of catalytic sites (Figure 5k,l). In contrast, as the hydrophobicity increases, the voltage and capacity of the Zn‐Co battery decrease due to the reduced contact area between the electrode and the electrolyte. By optimizing the active material loading and surface hydrophobicity, a Zn‐Co/air hybrid battery achieved an energy density of 936 Wh kg^−1^ (Figure 5m) and maintained the voltages in the range of 0.94–2.01 V for 400 cycles.

**Figure 6:**
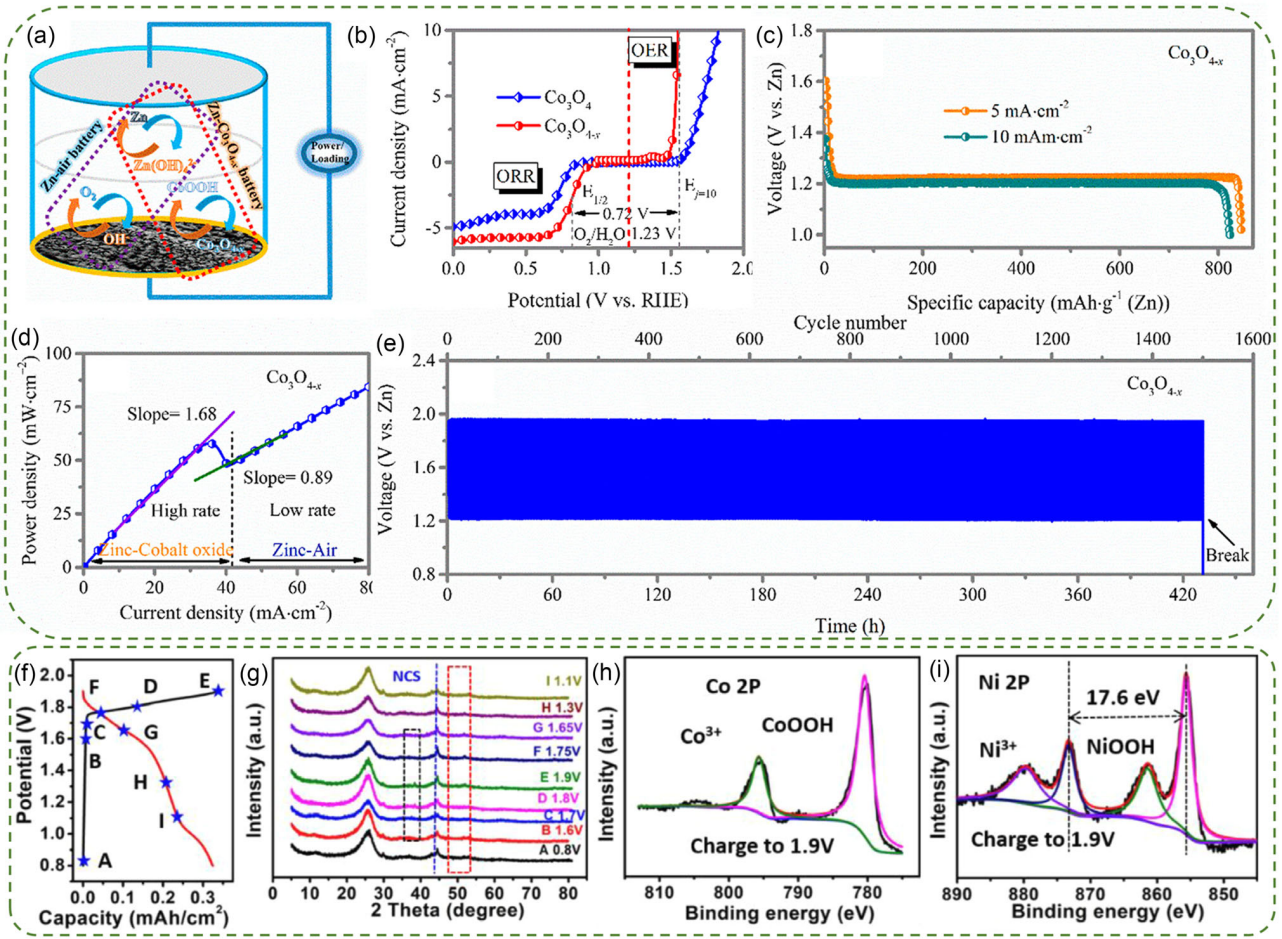
a) Schematic illustration of Zn‐Co/air hybrid battery. b) LSV curves of pristine Co_3_O_4_ and Co_3_O_4−*x*_ for OER and ORR overall polarization. c) Full discharging profiles recorded at 5 and 10 mA cm^−2^ and d) power density profile of hybrid battery using Co_3_O_4−*x*_ cathode. e) GCD cycling test recorded at 5 mA cm^−2^. Reproduced with permission.^[^ ^101^ ^]^ Copyright 2018, American Chemical Society. Reaction mechanism of the hybrid batteries. f) GCD profile, g) ex situ XRD data at different states, h,i) XPS of different elements when charged to 1.9 V. Reproduced with permission.^[^ ^105^ ^]^ Copyright 2021, Elsevier.

Abundant oxygen vacancies in Co_3_O_4_ greatly enhance its electrocatalytic performance for ORR and OER by increasing the number of electrochemically active sites. The bandgap defects in Co_3_O_4_ caused by these vacancies can significantly increase the electrical conductivity by facilitating the generation of two readily‐excited electrons.^[^ ^98^, ^99^, ^100^ ^]^ Zhi et al. introduced oxygen vacancies to cobalt oxide (denoted as Co_3_O_4−*x*_) using Ar‐plasma treatment (**Figure** **6**a).^[^ ^101^ ^]^ This treatment not only enhances the reversible redox reaction of Co (Co‐O ↔ Co‐O‐OH) but also results in good ORR and OER activities (ORR *E*_1/2_ of 0.84 V, and OER *E*_j10_ of 330 mV) (Figure 6b). The Zn‐Co/air hybrid battery with Co_3_O_4−*x*_ catalyst exhibited a power density of 3,200 W kg^−1^, an energy density of 1,060 Wh kg^−1^, and long‐cycling stability of 440 h for 1,500 cycles at 5 mA cm^−2^ (Figure 6c–e). Additionally, an oxygen vacancy‐rich Co_3_O_4_‐based cathode material (O‐Co_3_O_4_@MCN) was fabricated using Co‐based 1D coordination polymer as a precursor, which exhibited even distribution of Co_3_O_4_ particles ranging in size from 20 to 35 nm in the mesoporous N‐doped carbon matrix, resulting in enhanced activities of redox reaction activity and ORR/OER.^[^ ^102^ ^]^ Therefore, the hybrid battery with O‐Co_3_O_4_@MCN demonstrated a specific capacity of 790 mAh g^−1^, an energy density of 928 Wh kg^−1^ and a GCD stability of 300 h.

The incorporation of other transition metals such as Ni, Mn, and Cu into the Co_3_O_4_ lattice presents a feasible strategy for constructing high‐performance hybrid batteries.^[^ ^103^, ^104^ ^]^ Cui et al. selected porous NiCoSe_2_@NiOOH/CoOOH heterostructures (NCS@NCH) as cathode material.^[^ ^105^ ^]^ The phase change occurring during the charging and discharging process was detected by ex situ XRD measurement (Figure 6f,g). Gradually emerging peaks corresponding to NiOOH and CoOOH, especially at 1.8 and 1.9 V (Co(OH)_2_ + OH^−^ ↔ CoOOH + H_2_O + e^−^; Ni(OH)_2_ + OH^−^ ↔ NiOOH + H_2_O + e^−^), could be observed during charging. And disappearance of these peaks during discharging indicates the reduction of Co^3+^ and Ni^3+^. The formation of NiOOH and CoOOH at 1.9 V during charging was further confirmed by the Co 2*p* and Ni 2*p* XPS spectra (Figure 6h,i). Therefore, the hybrid battery exhibited an energy density of 944.8 Wh Kg^−1^, along with good cycling life in the sealed condition (105.1% after 4000 cycles) and open‐air state (100 h at 4 mA cm^−2^). Defect‐rich NiCo_2_O_4−*δ*_@C bubbles (DBHF) were also synthesized as the cathode catalyst.^[^ ^106^ ^]^ The battery assembled using the DBHF exhibited a steady voltage platform at 1.21 V during the initial discharging process, indicating a typical ORR process. Subsequently, during the charging process, two distinct voltage platforms at about 1.85 and 2.05 V were observed, corresponding to the M‐O oxidation and OER, respectively. In the second discharging, two voltage platforms emerged at 1.74 and 1.21 V, representing the reduction reaction of M‐O‐OH and ORR, respectively. Therefore, the hybrid battery displayed a capacity of 785 mAh g_Zn_^−1^ at 2 mA cm^−2^, a power density of 81.4 mW cm^−2^, and a high efficiency of 72%. The incorporation of Mn in the mixed valence states of Mn^3+^ and Mn^4+^ into the spinel structure of Co_3_O_4_ was found to enhance catalytic performance through the strengthening of C‐O‐metal and N‐O‐metal bonding.^[^ ^107^ ^]^ Zou et al. employed a facile hydrothermal method to prepare a self‐supported MnCo_2_O_4_ spinel electrode with a porous nanofiber morphology.^[^ ^108^ ^]^ The MnCo_2_O_4_ electrode exhibited a good electrochemical active surface area and suitable Co_2*p*_/Co_3*p*_ ratio, contributing to its bifunctional oxygen electrocatalytic performance and metal ion redox reaction activities. The assembled hybrid battery demonstrated an output voltage of 1.7 V, a power density of 67.9 mW cm^−2^, and a charge/discharge efficiency of 86.2%.

#### 3.1.3 Zn‐Ag/Air Hybrid Batteries

**Figure 7:**
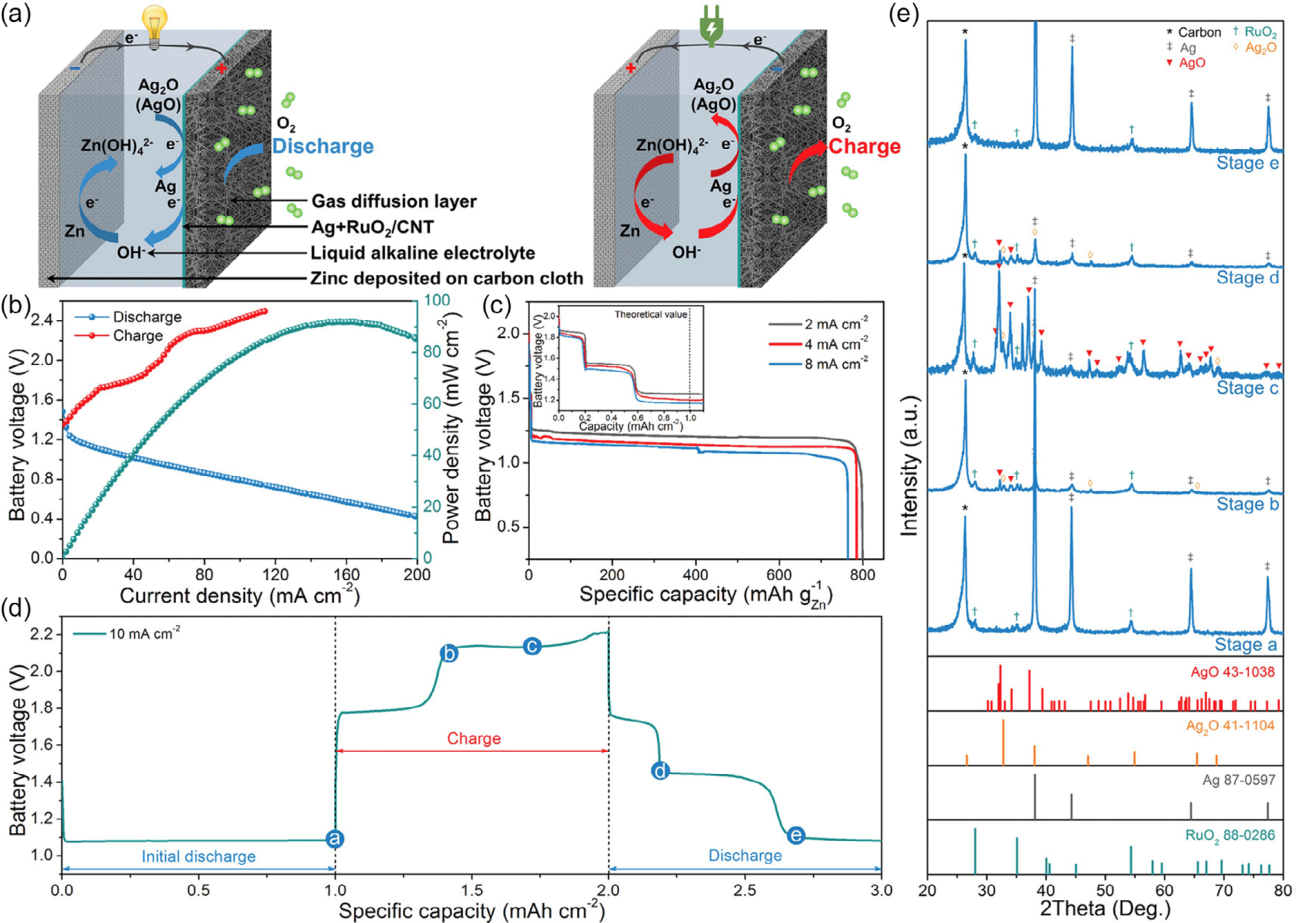
Electrochemical evaluation of Zn‐Ag/air hybrid battery. a) Schematic of the hybrid battery structure with proposed electrochemical processes during discharge and charge. b) Discharge and charge polarization and the corresponding power density. c) Galvanostatic discharge profiles at the current densities of 2, 4, and 8 mA cm^−2^ after charge polarization, the inset shows the initial voltage profiles. d) Initial discharge–charge–discharge voltage profiles at 10 mA cm^−2^. e) XRD patterns of the electrode at different states. Reproduced with permission.^[^ ^115^ ^]^ Copyright 2018 American Chemical Society.

Zn‐Ag batteries have gained wide application due to their intrinsic safety and energy density of up to 300 Wh kg^−1^.^[^ ^64^, ^109^, ^110^ ^]^ Typically, the Zn‐Ag battery demonstrates two distinct discharge voltage plateaus (1.80 and 1.55 V) when employing an Ag electrode. These voltage platforms corresponded to the reduction processes of Ag^2+^ to Ag^+^ and Ag^+^ to Ag, respectively.^[^ ^111^, ^112^, ^113^ ^]^ Among various potential metal elements such as Ni and Co for the ZMAHB system, Ag exhibits the highest electrical conductivity, greatly improving the cathode conductivity.^[^ ^53^, ^114^ ^]^ To address the insufficient OER activity of Ag, Ni et al. developed positive electrode materials by decorating carbon nanotubes with Ag and RuO_2_ nanoparticles (Ag + RuO_2_/CNT) for Zn‐Ag/air hybrid battery (**Figure** **7**a).^[^ ^115^ ^]^ The hybrid battery achieved a peak power density of 91.9 mW cm^−2^ at 157 mA cm^−2^ (Figure 7b,c), with corresponding specific capacity of 800 mAh g^−1^ at 2 mA cm^−2^. The charge and discharge mechanisms were confirmed through GCD curves and ex situ XRD analysis (Figure 7d,e). The voltage below 1.8 V and the second platform ≈2.0 V were associated with the oxidation of Ag (2Ag + 2OH^−^ ↔ Ag_2_O + H_2_O + 2e^−^; Ag_2_O + 2OH^−^ ↔ 2AgO + H_2_O + 2e^−^), and the voltages above 2.2 V matched OER process. During the charging process, the XRD peaks belonging to Ag significantly decreased, while new peaks related to Ag_2_O emerged. With increasing voltage, the Ag_2_O gradually oxidized into AgO. During discharging, three voltage steps were observed, corresponding to the reduction of AgO (AgO → Ag_2_O → Ag) and ORR process. In the discharging process, the AgO was gradually reduced to Ag_2_O and finally changed into Ag, indicating good reversibility. In a subsequent study, Chang et al. presented a Zn‐Ag/air hybrid battery using Ag nanoparticles covered in stainless steel wire screens.^[^ ^40^ ^]^ The assembled hybrid battery delivered two discharging platforms at 1.5 and 1.1 V, which were attributed to the reduction of Ag_2_O to Ag and Ag‐assisted ORR, respectively. The battery maintained a cycling life of 1,700 cycles (about 551 h) with the Coulombic efficiency retained higher than 85% at 20 mA cm^−2^.

**Figure 8:**
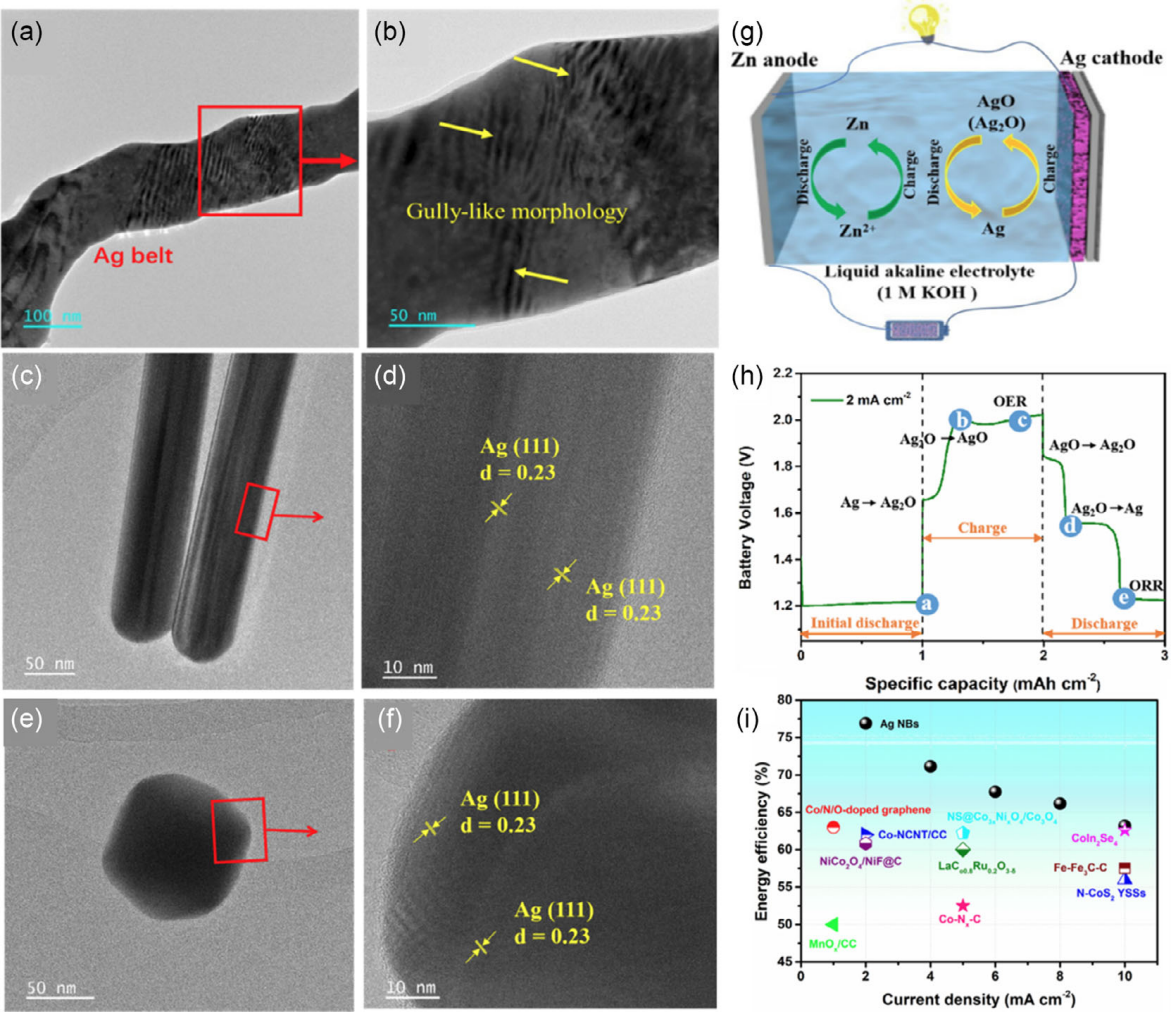
TEM and high resolution‐TEM of a,b) Ag nanobelts, c,d) Ag NWs, and e,f) Ag NPs. g) Schematic of a Zn‐Ag/air hybrid battery. h) Initial discharge–charge–discharge voltage profiles at 2 mA cm^−2^. i) The comparison of corresponding energy efficiency for the Ag NBs and the recently developed catalysts. Reproduced with permission.^[^ ^116^ ^]^ Copyright 2023, Elsevier.

The electrochemical activity of different morphologies of Ag nanomaterials, including Ag nanobelts (NBs), Ag nanoparticles, and Ag nanowires, was investigated by the Zhou group.^[^ ^116^ ^]^ They found that Ag NBs exhibited the highest conductivity due to their unique gully‐like morphology, raised Ag mounds, and superlattice fringes, as shown in **Figure** **8**a–f. These characteristics allowed Ag NBs to modify the surface electronic structure, enhancing catalytic performance and energy storage. When Ag NBs were employed as cathode material in the Zn‐Ag/air hybrid battery, three discharge voltage platforms were observed at 1.83, 1.55, and 1.23 V (Figure 8g,h), corresponding to the discharging process of the Zn‐Ag battery (AgO → Ag_2_O → Ag) and ORR. The hybrid battery delivered a high capacity of 496.92 mAh g^−1^ and a power density of 88.2 mW cm^−2^. The round‐trip energy efficiency was determined to be 76.9%, which is significantly higher than that of ZABs with bifunctional catalysts (Figure 8i).

Metallic Ag and its alloys with paragroup elements (such as AgCu, AgCo), featuring modified d‐band centers, have demonstrated attractive ORR activity in alkaline electrolytes, facilitating the complete reduction of O_2_ through a 4e^−^ pathway to OH^−^ with good stability.^[^ ^117^, ^118^ ^]^ Additionally, Ag exhibits reversible cathodic activity, making it a promising choice for Zn‐Ag batteries with high power output. For example, Peng et al. developed a self‐supporting cathode using a nanoporous Ag_2_Al intermetallic compound for the cathode material. This catalyst displayed good ORR performance and reversible redox activity, attributed to the presence of the abundant surface oxygen species, Ag‐Al intermetallic interaction, and np‐Ag_2_Al@AgAlO_*x*_ interface.^[^ ^119^ ^]^ The hybrid battery delivered a specific capacity of 3.23 mAh cm^−2^ and an energy density of 3.11 mWh cm^−2^. Similarly, Zhang et al. proposed the utilization of Ag‐embedded AlNiCoFeCr‐based high‐entropy oxides as electrode materials, taking advantage of their widely adjustable electronic structure.^[^ ^120^ ^]^ The resulting battery had an OCV of 1.528 V and an energy density of 810 Wh kg^−1^. Furthermore, the battery operated steadily under the current densities from 2 to 20 mA cm^−2^, with only a slight increase in the potential gap observed after 450 h of cycling.

#### 3.1.4 Zn‐Cu/Air Hybrid Batteries

**Figure 9:**
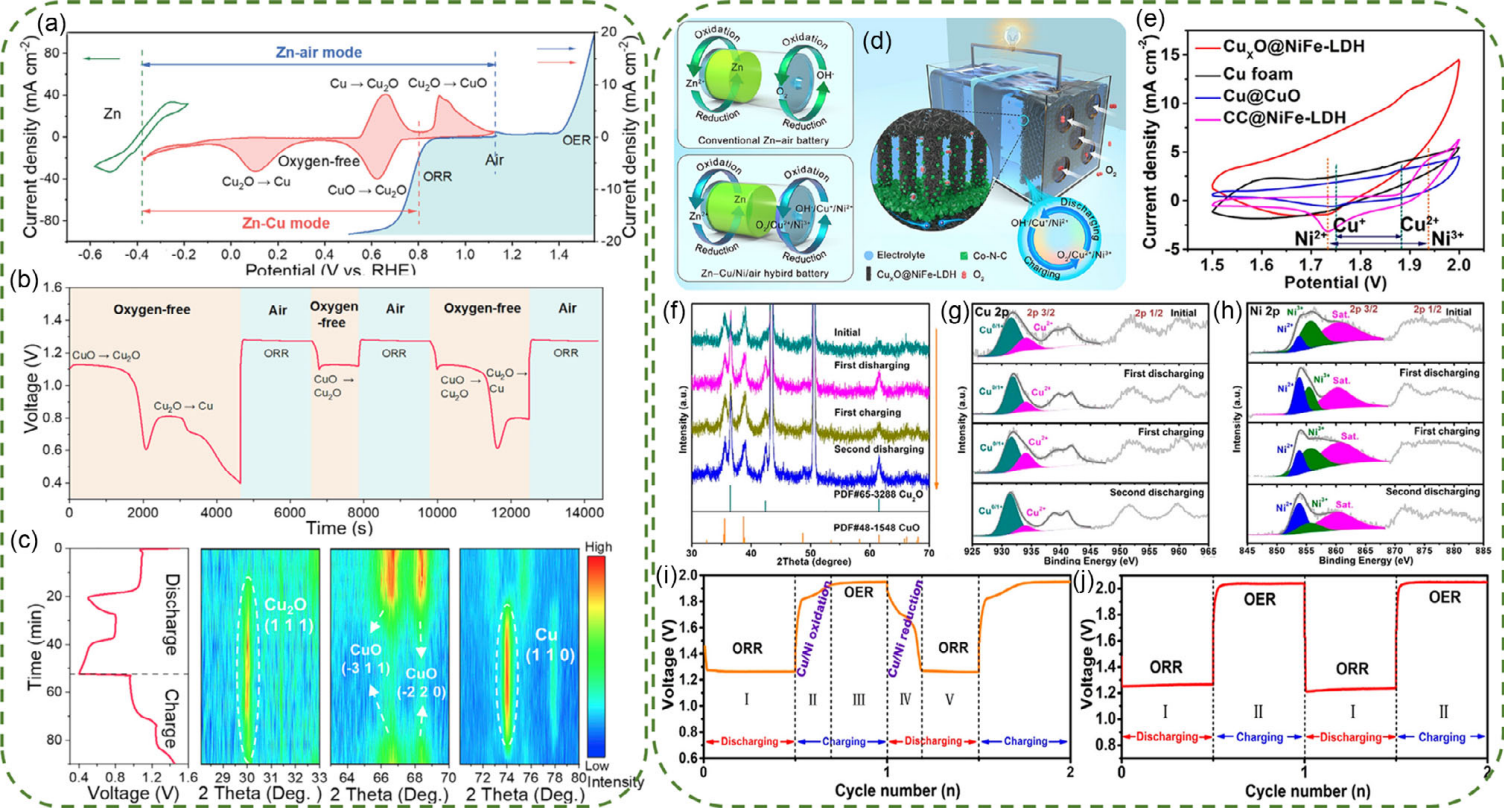
a) CV curves of Zn and CuO electrodes at 1 mV s^−1^ and the LSV curves of the ORR and OER under air. b) Discharge voltage curves at 2 mA cm^−2^ of the ZAB switching between the two conditions. c) The contour map of in situ XRD of the CuO electrode during the charging and discharging process. Reproduced with permission.^[^ ^123^ ^]^ Copyright 2022, Elsevier. d) Scheme of Zn‐Cu/Ni/air hybrid battery assembled by using Cu_*X*_O@NiFe‐LDH||Co−N−C as the air‐cathode. e) CV curves of different air‐cathode assembled batteries in air ambient. f) In situ XRD of Cu_*X*_O@NiFe‐LDH during charging and discharging processes. Ex situ XPS spectra of g) Cu 2*p* and h) Ni 2*p* of Cu_*X*_O@NiFe‐LDH after charging and discharging processes. The initial two cycles of GCD voltage profiles for i) Zn‐Cu/Ni/air hybrid battery and j) conventional ZAB. Reproduced with permission.^[^ ^124^ ^]^ Copyright 2022, American Chemical Society.

ZMAHBs using Ni, Co, and Ag‐based materials typically provide two or three discharge voltage stages, with the lowest voltage plateau typically representing the ORR process. For these types of hybrid batteries, the redox reaction in the ZMB occurs prior to the OER/ORR reaction in the ZAB. Consequently, the discharge voltage associated with the Zn‐M reaction is higher compared to that of the Zn‐air reaction segment.^[^ ^121^ ^]^ In other words, when the battery transitions to an anaerobic state, its operating voltage drops rapidly until it eventually fails due to the lack of reactants (O_2_). While these types of batteries are capable of functioning in oxygen‐free to oxygen‐feeding environments, they are unable to work when there is a reverse change in the atmospheric conditions. To overcome this limitation, Shang et al. propose a Zn‐Cu/air hybrid battery using CuO as cathode, based on the alkaline Zn‐Cu battery with a discharge voltage of 0.76 V.^[^ ^122^ ^]^ Copper‐based materials (such as Cu_2_O, CuO) exhibit catalytic performance for ORR in metal‐air batteries and undergo redox reactions to form Cu_2_O/Cu in Zn‐Cu batteries, enabling operation under both open and oxygen‐free environments.^[^ ^123^ ^]^ The electrochemical reaction system was further explored through the LSV curves presented in **Figure** **9**a. Two sets of redox peaks of CuO electrode were found at 0.11/0.66 V (Cu/Cu_2_O) and 0.62/0.89 V (Cu_2_O/CuO), respectively. The ORR catalyzed by CuO occurs when operated under air conditions, facilitating the ZAB mode. After transferring to an oxygen‐free environment, the ORR ceases due to the lack of O_2_, while CuO begins to perform a reduction reaction, causing a decrease in potential. Based on the proposed 2e^−^ transfer mechanism (Cu^2+^/Cu), the two reduction peaks can be tentatively attributed to the reduction of CuO to Cu_2_O and Cu_2_O to Cu, respectively. This reduction process gives rise to the two discharge voltage platforms, as depicted in Figure 9b. Therefore, when the battery is consumed in an oxygen‐free environment, it can still work under air and the discharge potential rises to 1.3 V. After operating under the air for ≈1,900 s, the hybrid battery could quickly turn to Zn‐Cu battery mode and continue discharging, verifying the rapid switchability. To further investigate the phase change during the charging and discharging process, in situ XRD was conducted, as demonstrated in Figure 9c. The electrode only shows two peaks corresponding to CuO and when the discharging process begins, the peak intensity of CuO gradually decreases with the formation of Cu_2_O, suggesting the transformation from CuO to Cu_2_O (2CuO + 2e^−^ ↔ Cu_2_O). As the charging process progressed, the peak intensity of Cu_2_O slightly decreased, and the peaks corresponding to Cu intensified, indicating the reduction from Cu_2_O to Cu (Cu_2_O + 2e^−^ ↔ 2Cu). The phenomenon during charging was the opposite, demonstrating the oxidation process of Cu → Cu_2_O → CuO.

By combining multiple redox pairs within the charge/discharge voltage range, the capacity and energy efficiency of the hybrid battery can be increased without enhancing the voltage gap. Zhang et al. proposed a hybrid battery system that integrates Zn‐air and Zn‐Cu/Zn‐Ni to achieve higher energy efficiency and stability (Figure 9d).^[^ ^124^ ^]^ The Ni^2+^/Ni^3+^ and Cu^+^/Cu^2+^ pairings were utilized, and Cu_*X*_O@NiFe‐LDH was chosen as the cathode electrode for the hybrid battery. The CV curves (Figure 9e) and GCD voltage profiles (Figure 9i,j) displayed that the hybrid battery had a smaller charging plateau and a lower voltage gap than traditional ZAB (RuO_2_ + CoNC). During the first discharging process (process I), mainly driven by ORR originating from Co‐N‐C, a discharge plateau of 1.27 V was achieved. Process II involved transformation from Cu‐O‐Cu to Cu‐O and Ni‐O to Ni‐O‐O‐H, resulting in a charging plateau of 1.8–1.9 V. Process III with a charging plateau of ≈1.95 V was assigned to OER. Finally, a discharging plateau of ≈1.7 V was achieved by the reduction of Cu‐O to Cu‐O‐Cu and Ni‐O‐O‐H to Ni‐O. In situ XRD analysis was used to characterize the phase changes, and peak intensity of Cu_2_O and CuO increased significantly after the discharging process and decreased after the first charging process (Figure 9f), suggesting the conversion between Cu^+^ and Cu^2+^. The ex situ XPS further confirmed the increased Cu^+^/Ni^2+^ contents and decreased Cu^2+^/Ni^3+^ contents after discharging (Figure 9g,h), suggesting the transformation from CuO/NiOOH to Cu_2_O/NiO. The inevitable oxidation of the catalyst during the ex situ characterization process in ambient air can lead to potential inaccuracies, and it is strongly recommended that follow‐up work uses a comprehensive approach, such as the combination of XRD and Raman, to validate the results obtained. Thus, the hybrid battery endowed an energy density of 940 Wh kg^−1^ and a lifetime of 500 h with an energy efficiency of 79.6% at 10 mA cm^−2^.

#### 3.1.5 Zn‐Mn/Air Hybrid Batteries

**Figure 10:**
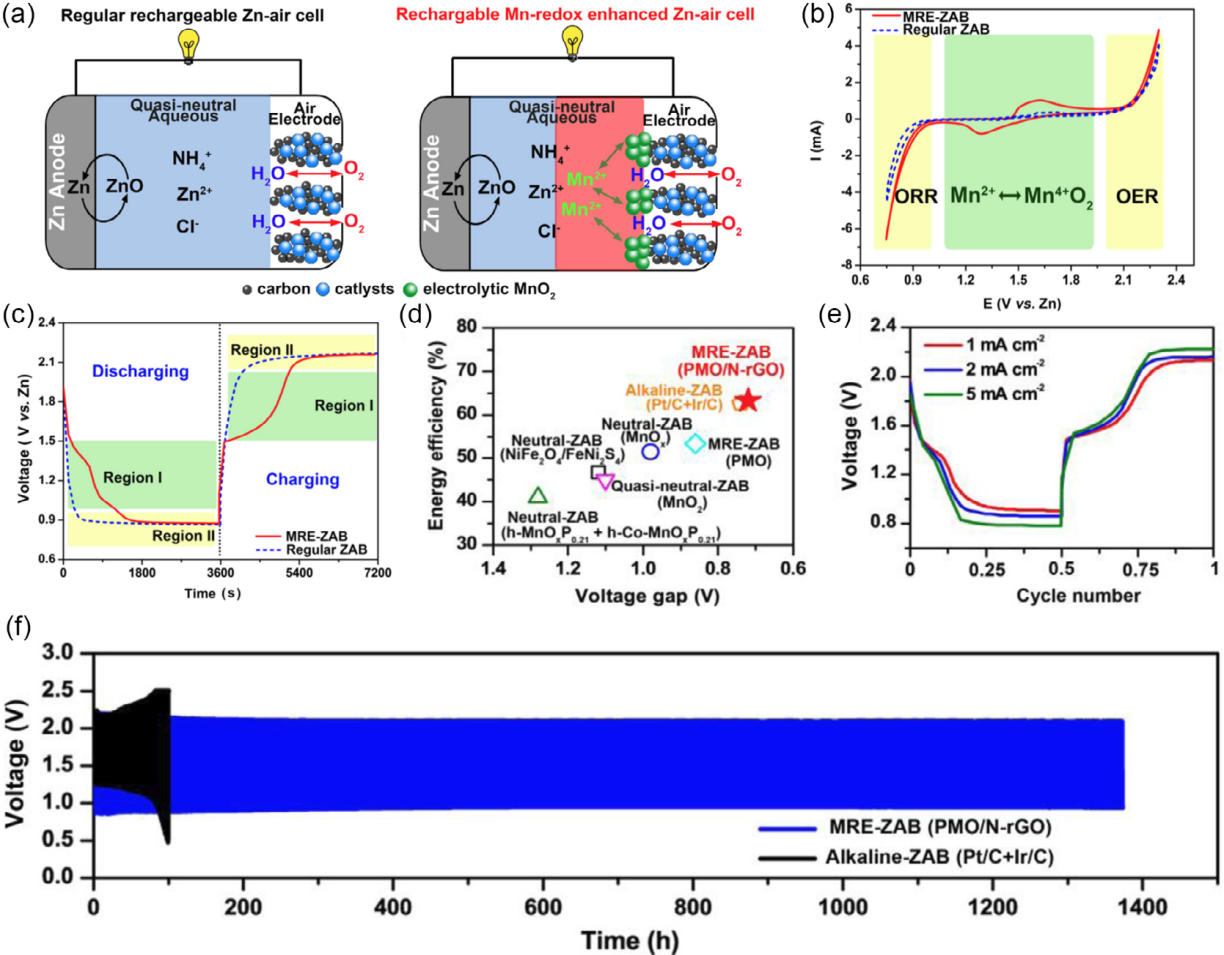
a) Schematic of a regular rechargeable ZAB with quasi‐neutral electrolyte and MRE‐ZAB with a quasi‐neutral Mn^2+^‐containing electrolyte. b) CV of quasi‐neutral MRE‐ZAB (red solid curve) and regular quasi‐neutral ZAB (blue dashed curve) at 0.2 mV s^−1^. c) GCD curves of quasi‐neutral MRE‐ZAB (red solid curve) and regular quasi‐neutral ZAB (blue‐dashed curve). d) Comparison of quasi‐neutral MRE‐ZAB with PMO/N‐rGO against other ZABs. e) Performance of a quasi‐neutral MRE‐ZAB with PMO/N‐rGO at different current densities. f) Long‐term stability of MRE‐ZAB with PMO/N‐rGO compared to the stability of a reference alkaline ZAB with Pt/C‐IrO_2_. Reproduced with permission.^[^ ^127^ ^]^ Copyright 2020, Elsevier.

Extending the design of hybrid batteries to neutral/quasi‐neutral ZABs, with the aim of further improving the round‐trip energy efficiency (currently less than ≈45% at low current densities), poses a challenge. The MnO_2_/Mn^2+^ redox reaction in neutral aqueous solutions, specifically the reversible transition between soluble Mn^2+^ and solid MnO_2_, is well‐compatible with ZAB batteries.^[^ ^125^, ^126^ ^]^ The Lee group proposed an Mn‐redox enhanced quasi‐neutral ZAB (MRE‐ZAB), which couples the ZAB mode with complementary MnO_2_/Mn^2+^ redox reactions (**Figure** **10**a).^[^ ^127^ ^]^ The electrolyte of the battery consisted of 2 m NH_4_Cl, 0.2 m ZnCl_2,_ and 0.02 m MnSO_4_, while the air electrode utilized P‐modified MnO_2_ supported on N‐doped reduced graphene oxide (PMO/N‐rGO). Apart from the substantial currents resulting from ORR below 1.0 V and OER above 1.90 V, the CV curve (Figure 10b) showed that the MRE‐ZAB had an additional couple of redox peaks at 1.3/1.6 V, corresponding to the transformation between Mn^2+^ and Mn^4+^. The GCD profile of quasi‐neutral MRE‐ZAB was similar to that of alkaline ZABs (Figure 10c). The steep curves observed in region I (1.0–1.9 V) indicated continuous changes in composition (Mn^2+^_(aq)_ + 2H_2_O_(l)_ ↔ MnO_2(s, electrolytic)_ + 4H^+^ (*aq*) + 2e^−^). Region II was characterized by a discharge voltage platform at approximately 0.9 V and a charge voltage platform at around 2.1 V, representing the ORR and OER processes, respectively.^[^ ^128^ ^]^ The MRE‐ZAB employing the PMO/N‐rGO catalyst achieved a round‐trip energy efficiency of 63.2%, the highest reported to date among neutral/quasi‐neutral ZABs (Figure 10d). The hybrid battery exhibited favorable activity in terms of battery rate, and maintained continuous operation for approximately 1350 h with no noticeable degradation (Figure 10e,f), surpassing the longevity of alkaline ZABs (100–150 h).

### 3.2 ZXAHBs

During the charging process of a ZAB, the cathode electrode undergoes OER with a four‐step electron–proton coupling transfer.^[^ ^129^, ^130^ ^]^ This reaction maintains a high thermodynamic equilibrium potential of 1.23 V, leading to a relatively high charging voltage of ZABs (>2.0 V), and has caused several problems, including degradation of the cathode material, shortened battery life, and low energy density.^[^ ^131^, ^132^, ^133^ ^]^ To tackle these challenges, a novel type of ZXAHB has been proposed that uses alternative substances prone to oxidation such as KI, urea, and ethanol (**Table** **4**).^[^ ^134^, ^135^, ^136^, ^137^, ^138^ ^]^ The bifunctional cathode material commonly used in ZABs is retained in the ZXAHBs, but requires improved catalytic performance for the corresponding oxidation reaction.^[^ ^54^ ^]^ In the following section, we will discuss the latest advances, design concepts, and electrochemical performance of ZXAHBs in detail.

**Table 4:** Performances of the ZXAHBs based on different cathode materials

Battery types	Cathode materials	Electrolyte	Discharge voltage [V]	Charge voltage [V]	Capacity@j	Energy density@j	Power density@j	Cycling stability@j	Energy efficiency	References
Zn‐KI/air	Pt/C/RuO_2_	PAM/SA/KI	1.29	1.63	–	–	132 mW cm^−2^@165 mA cm^−2^	100 h@1 mA cm^−2^	80 %	[141]
Zn‐KI/air	Pt/C/RuO_2_	PAM‐CNF/KOH/KI	1.03	1.75	818 mAh g^−1^@2 mA cm^−2^	–	48 mW cm^−2^@90 mA cm^−2^	75 h@2 mA cm^−2^	74%	[140]
Zn‐KI/air	Pt/C	6 m KOH + 3 m KI + 0.2 m Zn(Ac)_2_	1.31	1.68	800 mAh g^−1^@5 mA cm^−2^	–	148.8 mW cm^−2^@250 mA cm^−2^	80 h@5 mA cm^−2^	76.5%	[47]
Zn‐KI/air	Pt/C/RuO_2_	KI‐PVAA‐GO	1.24	1.69	742 mAh g^−1^@2 mA cm^−2^	–	78.6 mW cm^−2^@120 mA cm^−2^	200 h@2 mA cm^−2^	73%	[54]
Zn‐KI/air	S‐doped C_3_N_4_	1 m ZnCl_2_ in ethylene glycol	1.0	1.6	–	–	–	1300 h@1 mA cm^−2^	56.03%	[162]
Zn‐ethanol/air	Co(OH)_2_@Ni(OH)_2_	6.0 m KOH + 1.0 m EtOH	1.12	1.9	–	–	–	100 h@10 mA cm^−2^	59%	[148]
Zn‐urea/air	Ni SAs‐NC	4 m KOH + 0.33 m urea	1.2	1.68	–	831 Wh kg^−1^	165 mW cm^−2^@250 mA cm^−2^	300 h@10 mA cm^−2^	71.8%	[49]
Zn‐urea/air	Co/CoSe_2_@CN_*x*_	6 m KOH + 0.33 m urea	1.1	1.74	800 mAh g^−1^@10 mA cm^−2^	916.85 Wh kg^−1^@10 mA cm^−2^	113.16 mW cm^−2^@200 mA cm^−2^	140 h@10 mA cm^−2^	62.1%	[154]
Zn‐urea/air	V10%‐Ni_5_P_4_	4 m KOH + 0.33 m urea	1.3	1.75	–	–	127 mW cm^−2^@180 mA cm^−2^	400 h@5 mA cm^−2^	74.2%	[151]

#### 3.2.1 Zn‐KI/Air Hybrid Battery

**Figure 11:**
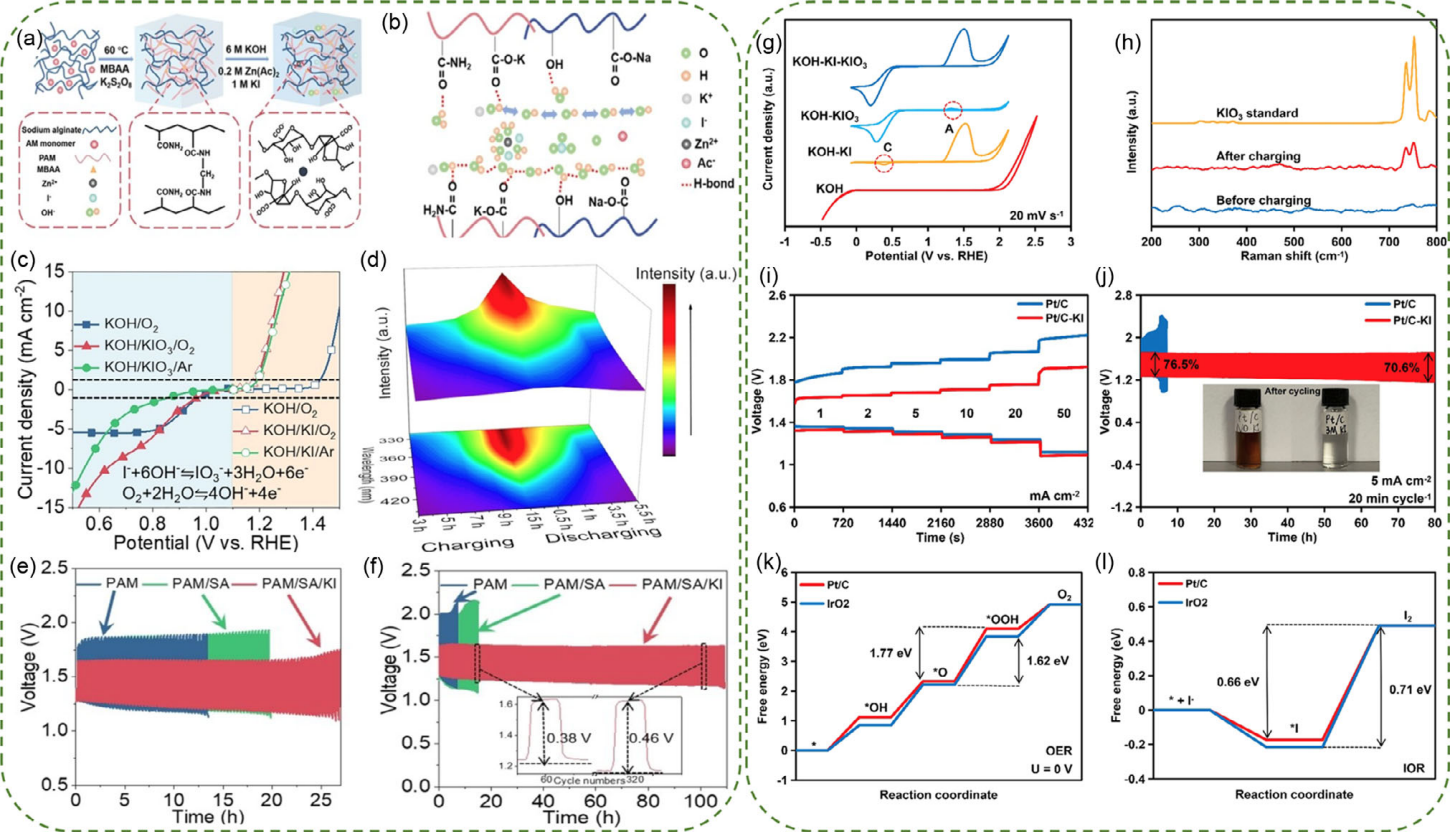
a) Synthetic process, b) schematic diagram of ion transport of PAM/SA/KI hydrogel. c) LSV curves of Pt/C + RuO_2_ composites in different O_2_‐ or Ar‐saturated electrolytes at 5 mV s^−1^. d) UV–vis spectra of KOH/Zn(Ac)_2_/KI solution for different charge/discharge times. e) GCD curves for PAM‐, PAM/SA‐, and PAM/SA/KI‐based batteries. f) Galvanostatic discharge curves and GCD curves of the initial PAM‐, PAM/SA‐, and PAM/SA/KI‐based batteries in the sealed configuration at 1 mA cm^−2^. Reproduced with permission.^[^ ^141^ ^]^ Copyright 2022, John Wiley and Sons. g) CV curves of the Pt foil in 1 m KOH, 1 m KOH + 0.5 m KI, 1 m KOH + saturated KIO_3_, and 1 m KOH + 0.5 m KI + saturated KIO_3_ solutions at 20 mV s^−1^. h) Raman spectra of the precipitates from the battery electrolyte before and after charging and the pure KIO_3_. i) Charge and discharge rate performances. j) GCD cycle performances at 5 mA cm^−2^. The inset shows the color change of the electrolytes after cycling k) OER and l) IOR free energy diagrams of the Pt/C and IrO_2_. Reproduced with permission.^[^ ^47^ ^]^ Copyright 2023, Elsevier.

The Zn‐KI/air hybrid battery has emerged as a promising candidate among hybrid battery systems. Iodine oxidation takes place when the battery is being charged, and oxygen reduction is the primary process during discharge. The addition of KI to the electrolytes transforms the OER process into an iodide oxidation reaction (IOR) with a lower oxidation potential, thereby preventing catalyst damage under high charging voltage.^[^ ^139^ ^]^ Since the IOR is a two‐electron process, it exhibits faster reaction kinetics than the sluggish OER process. This leads to a faster kinetic reaction rate, lower charging voltage, and higher energy efficiency of Zn‐KI/air hybrid batteries.^[^ ^140^ ^]^ Taking advantage of the above, Xia group reported a quasi‐solid Zn‐KI/air hybrid cell that modifies the charging reaction from OER to IOR by immersing the synthetic gel PAM/SA in a solution consisting of 6.0 m KOH, 0.2 m Zn(Ac)_2_ and 1.0 m KI (**Figure** **11**a,b).^[^ ^141^ ^]^ The reaction mechanism was investigated by a three‐electrode system in the different electrolytes (Figure 11c). The OER reached 1 mA cm^−2^ at 1.42 V (vs RHE), whereas iodide ion oxidation only 1.18 V to achieve 1 mA cm^−2^ (vs RHE) in the O_2_ or Ar‐saturated KOH/KI solution, indicating that iodide ion oxidation is the primary reaction during the charging process (I^−^ + 6OH^−^ → IO_3_^−^ + 3H_2_O + 6e^−^). In the O_2_‐saturated KOH/KIO_3_ condition, the LSV curves showed a distinct increase in current density compared to the O_2_‐saturated KOH electrolyte, providing evidence of the presence of ORR and IER simultaneously. In contrast, in the Ar‐saturated KOH/KIO_3_ condition, the current density of LSV curves was lower than that in O_2_‐saturated KOH electrolyte, suggesting that ORR reaction dominates in the discharging process.^[^ ^142^, ^143^ ^]^ The peak at 345 nm in ultraviolet‐visible spectra (Figure 11d) represented the iodate ions, further showing an increase adsorption during charging and a decrease adsorption during discharging. The PAM/SA/KI assembled battery exhibited a reduction in voltage gap, declining from 550 to 340 mV, accompanied by an energy efficiency of 80% based on the voltages of 1.29/1.63 V at current density of 1 mA cm^−2^ (Figure 11e). The existence of iodine species facilitated iodide/iodate redox reactions at cathode, enabling the hybrid battery to discharge and charge within a sealed system, effectively preventing electrolyte failure due to water loss. In a sealed system, the hybrid battery also displayed a long cycle stability of 110 h and an energy efficiency of 80% (Figure 11f).

The mechanisms of I^−^ oxidation pathway and its alternative to OER process in Zn‐KI/air hybrid batteries using Pt/C and IrO_2_ catalysts were further investigated by Ni group.^[^ ^47^ ^]^ The CV tests were performed in KOH solution containing KI, KIO_3_, or both (Figure 11g). The intense oxidation peak observed in KOH/KI condition corresponded to the oxidation reaction of I^−^, while high reduction peak in KOH/KIO_3_ electrolyte was ascribed to the reduction of IO_3_^−^. No other peaks appear in the CV curves, indicating that IO_3_^−^ was the final product of I^−^ oxidation, which was further confirmed by Raman studies (Figure 11h). The Zn‐KI/air hybrid battery based on Pt/C (Figure 11i) demonstrated a much narrower voltage gap than conventional ZABs. Additionally, the hybrid battery reached a steady voltage plateau of ≈1.63 V upon charging initiation and exhibited an energy efficiency of 76.5% at 5 mA cm^−2^. After 80 h, energy efficiency decreased to 70.6% (Figure 11j), which was higher than reported previously for traditional ZABs. The Gibbs free energy of the OER rate‐determining step for *O → *OOH of IrO_2_ (1.62 eV) was much smaller than that of 1.77 eV for Pt/C (Figure 11k,l), indicating that Pt/C has very poor OER activity. However, during IOR process, the rate‐determining step changed from *O → *OOH to *I → I_2_, with ΔG of 0.66 eV for Pt/C and 0.71 eV for IrO_2_, both of which were considerably smaller than those observed in OER. The I^−^ adsorption energy results demonstrated that Pt/C had a more intense interaction with I^−^ (*E*_ads_ = −0.936 eV) than IrO_2_ (−0.756 eV).

#### 3.2.2 Zn‐Ethanol/Air Hybrid Batteries

**Figure 12:**
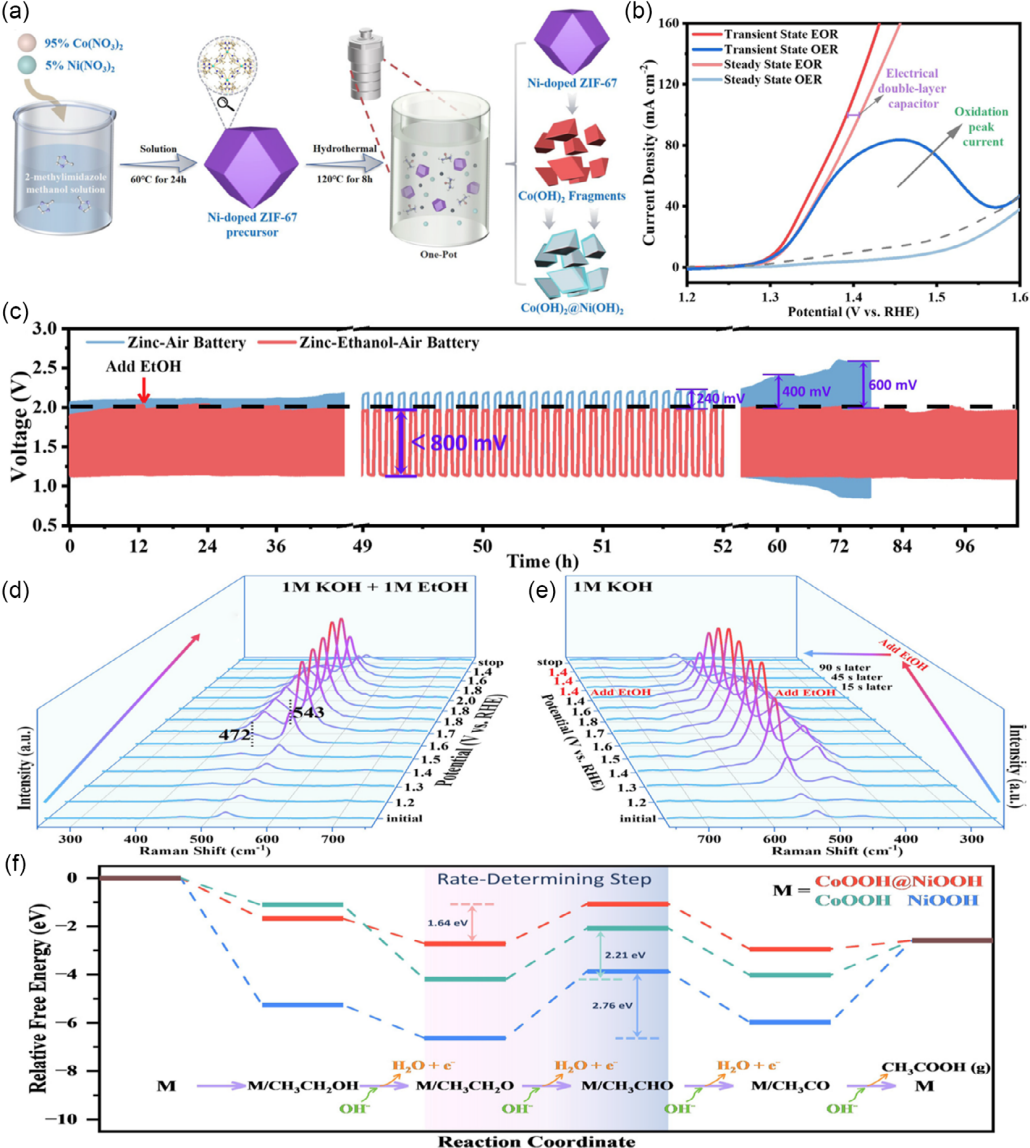
a) Schematic illustration of the preparation of the Co(OH)_2_@Ni(OH)_2_. b) Transient and steady‐state polarization curves of the EOR (in 1.0 m EtOH with 1.0 m KOH) and OER (in 1.0 m KOH) for Co(OH)_2_@Ni(OH)_2_. c) Cyclic measurements (current density of 10 mA cm^−2^) for ZAB and Zn‐ethanol/air battery. Potential dependent in situ Raman spectra for the Co(OH)_2_@Ni(OH)_2_ collected by multipotential steps in d) 1.0 m KOH with 1.0 m EtOH, and e) 1.0 m KOH. f) DFT‐calculated EOR energy profiles for CoOOH, NiOOH, and CoOOH@NiOOH. Reproduced with permission.^[^ ^148^ ^]^ Copyright 2022, Royal Society of Chemistry.

Recent studies have provided evidence that electrocatalytic oxidation reactions of certain small molecule organics (such as urea, alcohols, and KI) exhibit greater favorability compared to OER under alkaline electrolyte systems.^[^ ^144^, ^145^, ^146^, ^147^ ^]^ Hence, replacing anodic OER with these aforementioned oxidation reactions, which are characterized by more desirable kinetics and lower overpotentials, presents an opportunity to reduce the charging voltage and enhance round‐trip energy efficiency of ZABs. The construction of Zn‐ethanol/air battery involves the introduction of ethanol into the electrolyte, wherein the ethanol oxidation reaction (EOR) replaces the OER in alkaline conditions. This alteration eliminates the release of gas (O_2_/CO_2_) during the charging and mitigates catalyst shedding caused by O_2_ generated by OER. Consequently, this modification leads to a reduction in the charging voltage and enhances both the cycle life and energy efficiency of the battery. Additionally, the acetate generated by the oxidation of ethanol event can boost the conductivity of electrolytes in ZABs. According to the above characteristics, Sun et al. designed and fabricated an efficient catalyst for ethanol oxidation and integrated it into a Zn‐ethanol/air hybrid battery.^[^ ^148^ ^]^ As shown in **Figure** **12**a, the Co(OH)_2_@Ni(OH)_2_ heterostructure was synthesized by a one‐pot hydrothermal process and two‐step transformation, using a Ni‐doped ZIF‐67 precursor. The steady‐state LSV curves of EOR and OER (Figure 12b) indicated a much lower initial potential for EOR in comparison to OER, demonstrating good EOR activity of the Co(OH)_2_@Ni(OH)_2_ catalyst and its feasibility as OER substitute. Furthermore, a 4e^−^ EOR pathway of CH_3_CH_2_OH* ‐ CH_3_CH_2_O* ‐ CH_3_CHO* ‐ CH_3_CO* ‐ CH_3_COOH* was proposed. After that, the catalyst was integrated into a Zn‐ethanol/air hybrid battery with a 6 m KOH + 1 m EtOH electrolyte, and the charge‐discharge cycle tests were conducted at 10 mA cm^−2^. Figure 12c demonstrates that the charging voltage of hybrid battery is over 300 mV lower than that of ZABs at the same charging current density, with the charge‐discharge voltage gap remaining constant at 100 h. In situ Raman tests were performed to monitor the reaction process (Figure 12d,e), revealing weak peaks at 472 and 543 cm^−1^ before 1.5 V in the first test at 1 m KOH + 1 m EtOH. These weak peaks represent that β‐NiOOH, generated by oxidation of Ni(OH)_2_, reacted quickly with ethanol and could not be piled up in large quantities. As the voltage increases, it begins to accumulate. However, under 1 m KOH condition, large peaks at 472 and 543 cm^−1^ appeared at 1.3 V, proving slow OER reaction kinetics, and hindered consumption of β‐NiOOH, leading to rapid accumulation. Notably, when ethanol was added to the KOH, the Raman peak dropped sharply, suggesting that β‐NiOOH was rapidly consumed. These results confirmed that β‐NiOOH was the active species in both the EOR and OER, and the reaction kinetics of EOR was faster than that of OER. Density functional theory (DFT) calculations of EOR energy profiles (Figure 12f) revealed that M/CH_3_CHO was the most unfavorable intermediate during the ethanol upgrading conversion, theoretically suggesting that the EOR product was acetate instead of acetaldehyde.

Incorporation of 5–10% v/v ethanol into the electrolyte has also been shown to yield enhanced Zn dissolution and disrupt the passivation of the Zn anode.^[^ ^149^ ^]^ Experimental findings based on CV curves, electrochemical impedance spectroscopy analysis, and LSV measurements collectively demonstrated that the addition of a specific concentration of ethanol optimized the electrochemical performance. The galvanostatic discharge results revealed that the electrolyte containing 10% v/v ethanol displayed the highest electrochemical performance, resulting in a 30% increase in capacity and a 16% increase in energy density compared to the ethanol‐free KOH electrolyte.

#### 3.2.3 Zn‐Urea/Air Hybrid Batteries

**Figure 13:**
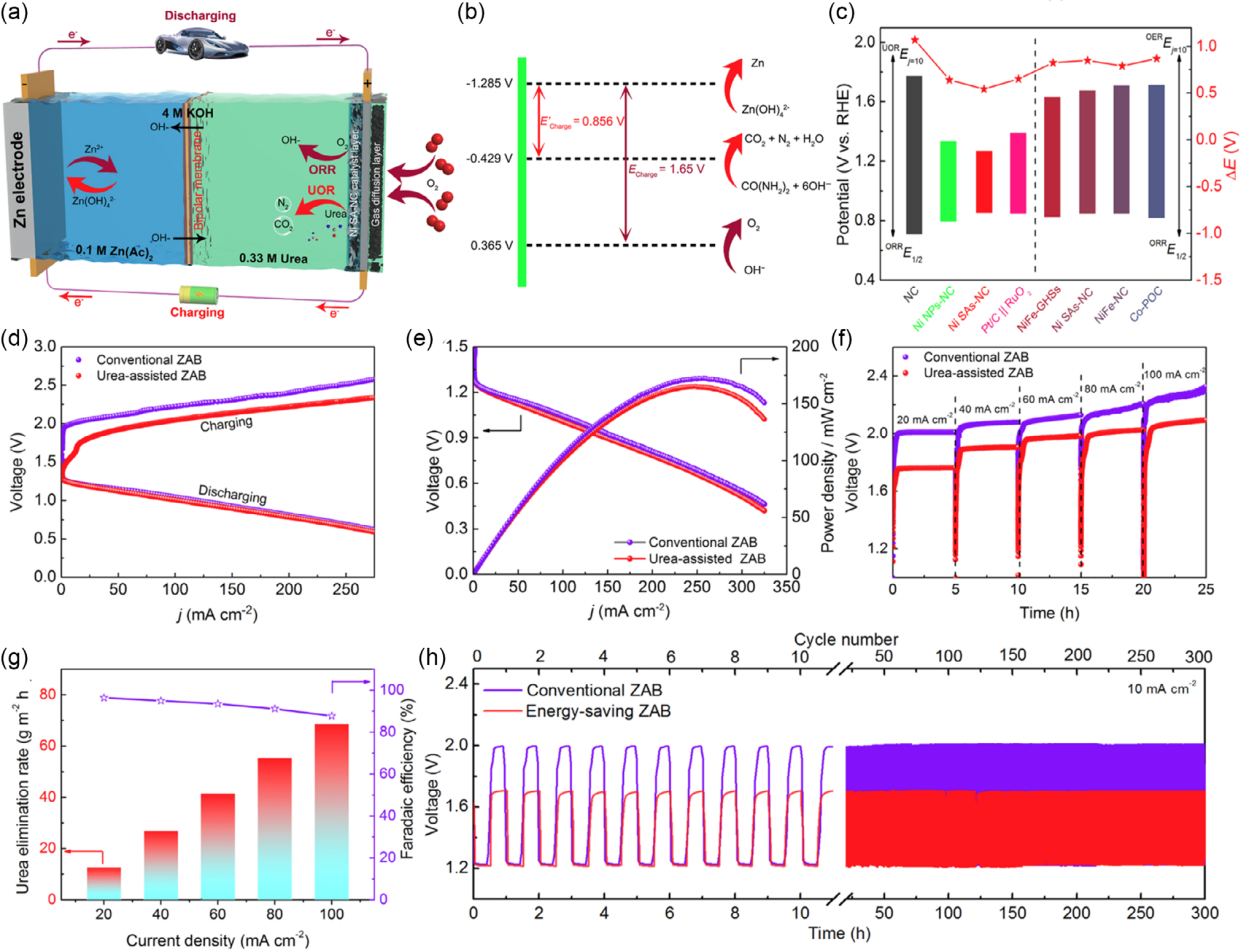
a) Schematic illustration of charging–discharging processes for Zn‐urea/air hybrid batteries. b) Comparison of the theoretical charging voltages of hybrid battery and ZABs. c) Comparison of ORR/UOR and ORR/OER bifunctional catalytic performance of different catalysts. d) Charging–discharging polarization curves, e) discharging polarization curves, and the corresponding power density plots of hybrid battery and ZAB. f) Galvanostatic charging curves of hybrid battery and ZABs at different current densities. g) Urea elimination rates and the corresponding faradaic efficiencies for the Ni SAs‐NC‐based hybrid battery at different current densities. h) GCD cycling curves of the hybrid battery and ZABs at 10 mA cm^−2^. Reproduced with permission.^[^ ^150^ ^]^ Copyright 2022, Elsevier.

Urea oxidation reaction (UOR) presents an appealing alternative to OER owing to its lower theoretical thermodynamic potential (−0.46 V vs SHE). Moreover, urea‐rich wastewater, such as urine, offers a plentiful and cost‐effective source for this reaction.^[^ ^150^ ^]^ The fundamental configuration and operating mechanism of Zn‐urea/air hybrid batteries were illustrated in **Figure** **13**a, which is composed of a Zn electrode with KOH and Zn(Ac)_2_ electrolyte as anolyte, an air cathode with KOH and urea electrolyte as catholyte. To prevent the undesired reaction of urea and zinc acetate under alkaline conditions, a bipolar membrane for ion transfer is required to separate the two electrolytes. Compared to OER process in traditional ZABs, the charging cycle of Zn‐urea/air hybrid batteries follows a different and more favorable course, primarily due to the low thermodynamic potential of UOR. The theoretical hybrid battery can be charged at a decreased voltage of 0.856 V (Figure 13b), which is 0.794 V below that of traditional ZABs.

To ensure the high‐performance operation of hybrid batteries, it is necessary to use advanced electrocatalysts for ORR and UOR. Zhang group reported a simple synthesis of a catalyst composed of Ni single atoms anchored on N‐doped carbon (Ni SAs‐NC), which exhibited a UOR potential of 1.39 V at 10 mA cm^−2^, lower than OER potential by 280 mV at the same current density.^[^ ^49^ ^]^ Thus, the potential gap (Δ*E*) of Ni SAs‐NC between *E*_j10_ for UOR and *E*_1/2_ for ORR is only 0.54 V (Figure 13c), signifying the better bifunctional activity among all the reported bifunctional materials. When Ni SAs‐NC was employed as the air cathode, the constructed hybrid batteries were demonstrated to have an energy density of 831 Wh kg^−1^ (Figure 13d–h) and a long‐term cycling life of 300 h. Moreover, the battery possessed a charging‐discharging voltage gap of 0.48 V and an energy conversion efficiency of 71.8%, representing a substantial improvement compared to conventional ZABs.

To enhance the UOR catalytic activity of materials, a particularly promising approach is to modulate the electronic structure through heteroatom doping. Cao et al. demonstrated the improved UOR activity of Ni_5_P_4_ by incorporating V atoms.^[^ ^151^ ^]^ Through a solvothermal‐phosphorization process, amorphous V‐doped Ni_5_P_4_ microflowers (V10%‐Ni_5_P_4_) with an optimized electronic structure were prepared. The V10%‐Ni_5_P_4_ microflowers, with V atoms incorporated, were assembled by much thinner and denser nanosheet units compared to Ni_5_P_4_ counterparts. The catalyst offers more active sites, improved mass transfer, and higher wettability because of this unique structure. The V10%‐Ni_5_P_4_ microflowers exhibited UOR performance of 282 mA cm^−2^ at 1.6 V (vs RHE). Besides, the Zn‐urea/air hybrid batteries had a larger OCV of 1.43 V and a smaller voltage gap than that Pt/C + IrO_2_‐based battery. The peak power density of V‐Ni_5_P_4_‐based hybrid battery (127 mW cm^−2^) was superior to that of Pt/C + IrO_2_ (111 mW cm^−2^) and Ni_5_P_4_‐based (112 mW cm^−2^) batteries.

Another widely employed approach in electrocatalysis is the construction of metal‐semiconductor heterojunctions to facilitate electron transfer rates at heterogeneous interfaces.^[^ ^152^, ^153^ ^]^ Wang et al. developed a core‐shell bifunctional ORR/UOR catalyst, consisting of the core of Co/CoSe_2_ heterojunction nanoparticles wrapped in a shell of N‐doped carbon (Co/CoSe_2_@CN_*x*_).^[^ ^154^ ^]^ The synergistic interaction of the Co/CoSe_2_ heterostructure promoted interfacial electron redistribution, thereby enhancing the bifunctional electrocatalytic activities for ORR and UOR. Additionally, the close integration of Co/CoSe_2_ with a hierarchically porous N‐doped graphitized carbon provided abundant active sites, increased electrical conductivity, and improved structural stability. Utilizing these advantages, Co/CoSe_2_@CN_*x*_ exhibited a low potential gap of 0.54 V between ORR and UOR, and the hybrid battery with this catalyst showed a small charging‐discharging voltage gap of 0.72 V and a long‐term cycle life of 140 h at 10 mA cm^−2^.

## 4 Conclusions and Outlook

**Figure 14:**
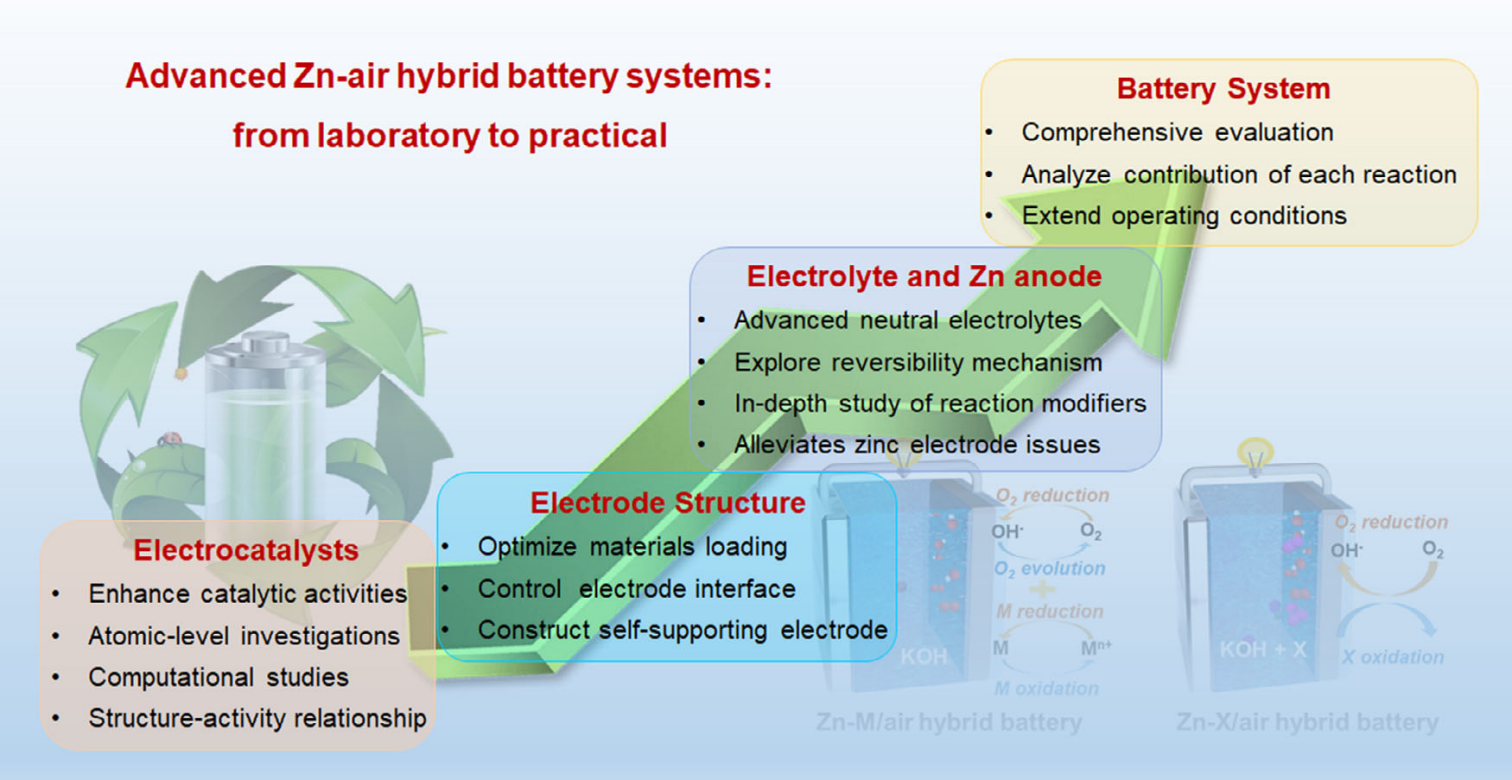
Perspectives on and opportunities for future research directions in ZAHB systems.

In this review, we have provided a comprehensive overview of recent advances in ZAHBs, encompassing both ZMAHBs and ZXAHBs. These innovative hybrid systems integrate the advantages of conventional ZABs with those of redox reactions, leading to reduced charging voltages, increased discharging voltages, as well as improved energy densities and efficiencies. ZMAHBs (M = Ni, Co, Ag, Cu, and Mn) integrating a ZMB with a ZAB enable a single cell to simultaneously achieve high energy densities and discharging voltages. Moreover, ZXAHBs are constructed by introducing easily oxidizable species (such as KI, ethanol, and urea) into the electrolyte, and by employing these oxidation reactions with lower oxidation potentials as a substitute for OER process, the energy efficiency can be significantly enhanced. This work provides an introduction to the classification, working principle, and recent works of these batteries, along with a summary of achieved electrochemical performances (**Figure** **14**).

First, a critical concern of the electrocatalytic activities at the cathode is often sluggish, thereby limiting the overall performance of ZAHBs. It is necessary to further improve the electrochemical performance of cathode materials. In the case of ZMAHBs, the cathode materials are used as the redox‐active media and electrocatalysts for oxygen electrolysis. Therefore, high capacities in the redox reaction and high catalytic efficiencies in ORR and OER are expected with the cathode materials. Similarly, for ZXAHBs, the obtained catalytic performance is the key to facilitating the corresponding oxidation reaction and ORR, thereby, further enhancing energy efficiency. Numerous strategies have been reported to optimize the performance of cathode materials, including adjusting the valence states of transition metals and oxygen, introducing dopant ions of a variety of species, and optimizing the morphology and microstructure of the materials. However, the electrocatalytic activity of currently reported catalysts is still unsatisfactory. More endeavors to investigate the cathode materials can be refined to the atomic level, taking into account the crystal phase orientation, chemical bond polarity, electronic structure of metal sites, interfacial location and spacing, and other atomic‐level properties, to realize various effects (electronic effects, ensemble effects, bifunctional effects, strain effects, etc.). To gain in‐depth understanding of the mechanisms related to specific atomic/interface structures, computational methods are invaluable tools. Based on computational predictions, the design of cathode materials can be optimized, and the structure‐activity relationship of the researched electrocatalysts can be reliably elucidated.

Efficient mass transfer of reactants such as oxygen and electrolytes to the electrode surface is critical to optimizing battery performance, and it is often influenced by electrode porosity, microscopic morphology, and so on. Therefore, the structural design of the electrode materials holds significant importance in achieving ideal battery performance. The ORR process primarily occurs at a triple‐phase interface consisting of the solid electrode, liquid electrolyte, and gaseous oxygen. Conversely, the OER and redox reactions of active materials take place at the two‐phase interface formed by the solid electrode and the liquid electrolyte. As a result, the construction of an air electrode structure becomes complicated, while a delicate balance between the three‐phase and two‐phase interfaces needs to be established. At present, more studies on the control of hydrophilicity and hydrophobicity of air electrode surfaces to regulate the catalytic reaction interface have been carried out in the field of ZABs, but the application of such research in hybrid batteries remains relatively lacking. Moreover, the cathode loading also significantly affects the activity of ZMAHBs. The increasing loading may lead to higher availability of reactants for the Zn‐M reaction, but much higher loading can also result in a thicker increase of the electrode thickness and the increased resistance to oxygen transport, which subsequently hampers the performance of the Zn‐air reaction. Therefore, to achieve better charge–discharge performance, it is essential to optimize both the engineering electrode interface and the loading of active species. Integrated self‐supporting electrodes based on current collectors hold promising potential for broader applications in the future, and further research on their practical implementation is highly desirable.

The appropriate electrolyte concentration is a vital optimization step toward attaining the desired power and energy density in ZAHBs.^[^ ^155^ ^]^ The electrolyte concentration in a battery directly affects its conductivity, ion transport, and other critical factors, as well as the formation and stability of the electrode‐electrolyte interface, thereby altering the electrocatalytic overpotential of the electrode and impacting battery performance.^[^ ^156^ ^]^ Further research efforts are expected to develop novel electrolytes, which arguably have a greater impact on the fundamental mechanisms of ZAHBs. Although there is a growing number of studies on quasi‐neutral electrolytes, such as the water‐in‐salt systems, their specific applications in ZABs and ZAHBs are seldom investigated. A comprehensive understanding of the cathodic reversibility mechanism and redox chemistry of oxygen pertaining to various types of electrolytes is imperative to propel their development. Some neutral electrolytes in batteries have been explored, such as Zn‐Mn/air hybrid batteries, and there is wide space for research on other neutral systems in the future. In addition, to enhance the performance of ZXAHBs, meticulous investigations of the precise role of each additive through in‐depth characterization and analysis are highly desired. In the case of ZXAHBs, the semireactions of charging and discharging processes are independent, and a cycle of reactants and products cannot be established. Once the additive modifiers are exhausted, the charging voltage will increase substantially, which results in a short cycle time for hybrid batteries and therefore hinders their commercialization. The development of efficient catalysts and the solution to the recycling problem for ZXAHBs become urgent matters to be addressed.

In contrast, it should be noted that the negative electrode, namely, the Zn electrode, also has a significant impact on the battery performance. The relevant problems such as dendrite formation, inadvertent migration, and ZnO passivation are closely associated with the performance degradation. Recently, several studies addressing these concerns have been reported, including the development of porous Zn structures with high surface area, encapsulation of Zn in 3D host materials, and direct physical inhibition/inhibition methods.^[^ ^157^, ^158^ ^]^ However, it is still necessary to develop efficient and low‐cost methods to alleviate the problem of Zn electrodes.

A comprehensive evaluation of the electrochemical performance of ZAHBs with advanced cathodes is essential. Previous studies have predominantly assessed the electrochemical performance of ZAHBs using the methods employed for ZABs. These methods involve evaluating GCD curves to measure the energy efficiency and cycle stability, and utilizing constant‐current discharge curves to measure the specific capacity and energy density. However, due to the combination of additional oxidation/reduction processes in ZAHBs, various assessment aspects need to be considered. The coexistence of supplemental redox reactions and oxygen electrocatalysis within ZAHBs closely links the contributions of these reactions to the discharge depth in the charge–discharge cycles, which therefore has an important impact on energy efficiency. Systematic investigations are required to deal with the additional contribution and in‐depth influences of additional reactions on battery performance. In addition, the performance of ZAHBs with semiopen systems is significantly influenced by the working conditions such as temperature and humidity. Several ZAB systems adapted to extremely high or low‐temperature reactions have been reported, but the influence of such extreme conditions on ZAHB performance has not been reported yet. Moreover, ZAHBs are mainly operated at low current densities at present, similar to conventional ZABs, and the performance at high current densities is a major challenge.^[^ ^159^ ^]^ Continued progress in catalytic materials, electrode designs, and advanced electrolytes are needed to improve the performance and wider applications of hybrid batteries in the future.

Due to the synergistic combination of fast redox reactions and high potentials, ZAHBs have demonstrated the promising potential to deliver high power density and fast charge–discharge rates. For example, Zn‐Ni/air hybrid batteries have a remarkable ability to charge at high rates without loss of discharge capacity and energy efficiency, a feature that reduces charging time and has immense potential for various future applications, including portable electronics and smart grid energy storage.^[^ ^43^ ^]^ Furthermore, quasi‐solid‐state batteries, inspired by recent advances such as sponge‐like electrodes and polymer gel electrolytes, have emerged as another promising prospect.^[^ ^160^ ^]^ Using gel electrolytes instead of aqueous electrolytes can make flexible batteries suitable for wearable electronics, such as smartwatches.^[^ ^40^ ^]^ Besides powering flexible devices, solid‐state batteries hold great promise for electric vehicle applications.^[^ ^120^, ^161^ ^]^ The development of ZAHBs for practical implementation is still in its early stages, and their current performance is more suitable for low‐power wearable devices, but their future is promising in the growing field of power batteries.

In conclusion, by optimizing the electrode materials, engineering the reaction interface, regulating the electrolytes and the Zn anode, and implementing effective operation management, ZAHBs can achieve high energy density and efficiency and have great potential for future applications. The review can provide valuable guidance for the development of ZAHB systems and be helpful for future research work.

## Conflict of Interest

The authors declare no conflict of interest.
